# Heterogeneity of Metabolic Vulnerability in Imatinib-Resistant Gastrointestinal Stromal Tumor

**DOI:** 10.3390/cells9061333

**Published:** 2020-05-26

**Authors:** Wen-Kuan Huang, Jiwei Gao, Ziqing Chen, Hao Shi, Juan Yuan, Huanhuan L. Cui, Chun-Nan Yeh, Robert Bränström, Catharina Larsson, Shuijie Li, Weng-Onn Lui

**Affiliations:** 1 Department of Oncology-Pathology, Karolinska Institutet, BioClinicum J6:20, Karolinska University Hospital, SE-17164 Solna, Sweden; wen-kuan.huang@ki.se (W.-K.H.); jiwei.gao@ki.se (J.G.); chen.ziqing@ki.se (Z.C.); hao.shi@ki.se (H.S.); catharina.larsson@ki.se (C.L.); 2 Division of Hematology-Oncology, Department of Internal Medicine, Chang Gung Memorial Hospital at Linkou, Chang Gung University College of Medicine, Taoyuan 33305, Taiwan; 3 Department of Cell and Molecular Biology, Karolinska Institutet, Solnavägen 9, SE-17165 Stockholm, Sweden; juan.yuan@ki.se; 4 Department of Medicine-Solna, Microbial Pathogenesis Unit, Karolinska Institutet, BioClinicum, SE-17164 Solna, Sweden; leah.cui@ki.se; 5 Department of Surgery, Chang Gung Memorial Hospital and GIST team at Linkou, Chang Gung University College of Medicine, Taoyuan 33305, Taiwan; yehchunnan@gmail.com; 6 Department of Molecular Medicine and Surgery, Karolinska Institutet, SE-17176 Stockholm, Sweden; robert.branstrom@ki.se; 7 Department of Microbiology, Tumor and Cell Biology, Karolinska Institutet, SE-17177 Stockholm, Sweden

**Keywords:** gastrointestinal stromal tumor, energy metabolism, mitochondrial biogenesis, imatinib resistance

## Abstract

Metabolic reprogramming is a hallmark of cancer cells in response to targeted therapy. Decreased glycolytic activity with enhanced mitochondrial respiration secondary to imatinib has been shown in imatinib-sensitive gastrointestional stromal tumors (GIST). However, the role of energy metabolism in imatinib-resistant GIST remains poorly characterized. Here, we investigated the effect of imatinib treatment on glycolysis and oxidative phosphorylation (OXPHOS), as well as the effect of inhibition of these energy metabolisms on cell viability in imatinib-resistant and -sensitive GIST cell lines. We observed that imatinib treatment increased OXPHOS in imatinib-sensitive, but not imatinib-resistant, GIST cells. Imatinib also reduced the expression of mitochondrial biogenesis activators (peroxisome proliferator-activated receptor coactivator-1 alpha (PGC1α), nuclear respiratory factor 2 (NRF2), and mitochondrial transcription factor A (TFAM)) and mitochondrial mass in imatinib-sensitive GIST cells. Lower TFAM levels were also observed in imatinib-sensitive GISTs than in tumors from untreated patients. Using the Seahorse system, we observed bioenergetics diversity among the GIST cell lines. One of the acquired resistant cell lines (GIST 882R) displayed a highly metabolically active phenotype with higher glycolysis and OXPHOS levels compared with the parental GIST 882, while the other resistant cell line (GIST T1R) had a similar basal glycolytic activity but lower mitochondrial respiration than the parental GIST T1. Further functional assays demonstrated that GIST 882R was more vulnerable to glycolysis inhibition than GIST 882, while GIST T1R was more resistant to OXPHOS inhibition than GIST T1. These findings highlight the diverse energy metabolic adaptations in GIST cells that allow them to survive upon imatinib treatment and reveal the potential of targeting the metabolism for GIST therapy.

## 1. Introduction

Gastrointestinal stromal tumor (GIST) is a mesenchymal tumor that frequently harbors *KIT* receptor tyrosine kinase mutations [1]. The majority of these patients benefit from imatinib treatment; however, a large proportion of patients develop imatinib resistance within two years [2]. The minimal benefit of sunitinib and regorafenib in imatinib-resistant patients highlights the need to explore novel resistant mechanisms.

Cancer cells are commonly characterized by intense aerobic glycolysis with a decrease in mitochondrial energy metabolism [3]. The metabolic adaptation to the toxic effects of targeted drugs has been shown to contribute to drug resistance [4,5,6,7]. In these models, resistant subsets of cancer cells rely on increased mitochondrial function and oxidative phosphorylation (OXPHOS). By contrast, a metabolic shift toward the Warburg effect has also been implicated in anticancer drug resistance [8,9]. Furthermore, cancer stem cells, a small subpopulation inherently resistant to cytotoxic challenge, often rely on glycolysis for cell growth [10]. These findings indicate that targeting context-dependent metabolic traits of resistant cancer cells provides a promising approach for overcoming drug resistance.

While GIST demonstrates intense glucose uptake and glycolysis activities, imatinib stress leads to metabolic reprogramming towards an enhanced mitochondrial respiratory capacity [11]. Imatinib combined with the inhibition of mitochondrial OXPHOS intensifies the efficacy of imatinib monotherapy. However, the energy metabolism in imatinib-resistant GIST remains unclear. Herein, we characterize the energy metabolism of imatinib-resistant GIST in comparison to imatinib-naïve GIST. We demonstrate the heterogenous energy metabolism of imatinib-resistant GIST cells. Furthermore, subsets of imatinib-resistant GIST cells are found to be more vulnerable to metabolic/energy stress than imatinib-sensitive GIST cells.

## 2. Materials and Methods

### 2.1. Clinical Samples

A total of 39 snap-frozen GIST tumors (from 20 untreated patients and 15 imatinib-treated patients) were used in this study. The details of treated cases, including 8 responding and 11 resistant tumors, have previously been described [12]. The clinical, genetic, and histopathological characteristics of all 35 cases are presented in Table S1. The samples were obtained from Karolinska University Hospital Biobank. All the samples had been collected with informed consent, and the study of the tissue materials was approved by the local ethical committee in Stockholm, Sweden.

### 2.2. Human GIST Cell Lines and Imatinib-Resistant Derivatives

The GIST 882 and GIST 48 were kindly provided by Dr. Jonathan Fletcher at Brigham and Women’s Hospital, Boston (MA, USA). The GIST T1 was purchased from Cosmo Bio Co. Ltd. (Tokyo, Japan). The GIST 882 cells were cultured in Roswell Park Memorial Institute (RPMI) 1640 media supplemented with 15% fetal bovine serum. The GIST T1 cells were cultured in Dulbecco’s Modified Eagle Medium (DMEM) supplemented with 10% fetal bovine serum. The GIST 48 cells were grown in F-10 media supplemented with 15% fetal bovine serum, 2.5 µg/mL of MITO plus serum extender (Corning, New York, NY, USA), and 5 µg/mL of bovine pituitary extract (Thermo Fisher Scientific, Waltham, MA, USA). All the cell lines were maintained in a humidified 37 °C incubator with 5% CO_2_. The two imatinib-sensitive cell lines, GIST 882 and GIST T1, were used to generate imatinib-resistant derivative cell lines, GIST 882R and GIST T1R, by continually exposing them to 1 µM of imatinib for at least 8 months. The GIST 48 cell line is an established imatinib-resistant cell line [13]. The cell lines were verified by short tandem repeat profiling performed by the National Genomics Infrastructure in Uppsala (SciLifeLab, Uppsala University, Sweden). The resulting genotypes are detailed in Table S2.

### 2.3. GEO Dataset Analysis

We extracted the microarray gene expression data for 15 imatinib-resistant GISTs (accession number GSE132542) deposited in the National Center for Biotechnology Information (NCBI) Gene Expression Omnibus (https://www.ncbi.nlm.nih.gov/geo/query/acc.cgi?acc=GSE132542). The samples were clustered based on Euclidean distances using the normalized mRNA expression levels of gene sets for OXPHOS and glycolysis from the Molecular Signature Database (Broad Institute, Version 7.0). The differentially expressed genes between cluster I and II were analyzed using the limma package in R (version 3.42.0), and differentially expressed genes with a false discovery rate of <0.2 were used for a clustering analysis using the Complex Heatmap (v1.6.0) R package (v3.6.1).

### 2.4. Flow Cytometry for Mitochondrial Mass Measurement and Reactive Oxygen Species (ROS)

GIST 882, 882R, T1, and T1R cells seeded in 6-well plate at a 70% confluency were treated with 1 µM of imatinib. After 48 h, the cells were harvested, washed, and stained with MitoTracker Green FM and a LIVE/DEAD Viability Kit (Thermo Fisher Scientific, Waltham, MA, USA) according to the manufacturer’s instructions. The mean Mitotracker green fluorescence intensity of the live cells was measured by a NovoCyte Flow Cytometer (ACEA Biosciences, San Diego, CA, USA), and the data were analyzed using FlowJo software (FlowJo, LLC, Ashland, OR, USA).

The intracellular ROS levels of each cell line were measured using 2′,7′-dichlorofluorescin diacetate (DCFH-DA, Sigma-Aldrich, Darmstadt, Germany). The cell pellets were collected, washed with phosphate-buffered saline (PBS) and resuspended in PBS containing 5 µM of DCFH-DA. After incubation in the dark for 30 min at 37  °C, the residual DCFH-DA was removed by washing with PBS. The ROS content was measured by a NovoCyte Flow Cytometer (ACEA Biosciences, San Diego, CA, USA) using a filter with an excitation/emission = 485/535 nm.

### 2.5. Glucose Uptake Assay 

The glucose uptake was measured using the 2-NBDG Glucose Uptake Assay Kit (Abcam, Cambridge, UK) following the manufacturer’s recommendations. Briefly, after 1 h of glucose deprivation, the cells were incubated with a glucose-free medium containing a fluorescently-labeled deoxyglucose analog, 2-(*N*-(7-nitrobenz-2-oxa-1,3-diazol-4-yl)amino)-2-deoxyglucose (2-NBDG, 100 µg/mL), for 45 min at 37  °C. The cells were washed with a Cell-Based Assay Buffer twice to remove the excess 2-NBDG and then detected by a NovoCyte Flow Cytometer (ACEA Biosciences, San Diego, CA, USA), using a filter with an excitation/emission = 485/535 nm.

### 2.6. Immunofluoresence Analysis

The GIST 882, 882R, T1, and T1R cells were plated on a coverslip with 2 × 10^5^ cells in a 6-well tissue culture plate. After treatment with or without 1 µM of imatinib for 48 h, the cells were fixed in cold 4% paraformaldehyde for 15 min and permeabilized with 0.1% Triton 100 in cold PBS for 15 min. Then, the cells were incubated with the primary antibody peroxisome proliferator-activated receptor coactivator-1 alpha (PGC1α) (dilution 1:250, Sigma-Aldrich, #ST1202) at 4 °C overnight, followed by incubating with anti-mouse Alexa 488-conjugated secondary antibody (Thermo Fisher Scientific, Waltham, MA, USA) for 1 h. The coverslips were mounted in VectaShield mounting media containing 4′,6-diamidino-2-phenylindole (DAPI) for a nuclear counterstain (Vector Laboratories, Burlingame, CA, USA). Images were captured using a Zeiss fluorescence microscope (Axio Observer 7, Carl Zeiss, Jena, Germany) and ZEN software (Carl Zeiss). The quantification of the PGC1α-stained cells was calculated based on the mean fluorescence intensities of the images that were captured from three different fields and normalized to their respective DAPI-stained nuclei.

### 2.7. Real-Time Glycolytic and Mitochondrial Respiration Rate Measurement

Seahorse XFe96 Extracellular Flux Analyzer (Agilent Technologies, Santa Clara, CA, USA) was used for the metabolic analyses. GIST 882 and 882R, T1 and T1R, and 48 cells were seeded in each well at a density of 50,000, 20,000, and 30,000 cells, respectively. The oxygen consumption rate (OCR) was measured under basal and stress conditions with sequential injections of 1 mM of oligomycin, 1.6 mM of carbonyl cyanide-4 (trifluoromethoxy) phenylhydrazone (FCCP), and 0.5 mM of antimycin/rotenone, following the manufacturer’s instructions of the XF Cell Mito Stress Test Kit (Agilent Technologies, Santa Clara, CA, USA). The extracellular acidification rate (ECAR) was determined at basal and stress conditions after the sequential addition of 10 mM of glucose, 1 mM of oligomycin and 100 mM of 2-deoxyglucose, following the manufacturer’s instructions of the XF Glycolysis Stress Test Kit (Agilent Technologies, Santa Clara, CA, USA). All the metabolic assays were normalized to the number of cells.

### 2.8. Cell Proliferation Assay

The GIST 882, 882R, or 48 cells were seeded at a density of 20,000 cells/well, and the GIS*T*-T1 or T1R cells were seeded at a density of 10,000 cells/well in 96-well plates. The cells were cultured with 3-bromopyruvate (3-BP), gossypol, antimycin A, oligomycin A, or dimethyl sulfoxide (DMSO)-only medium (Sigma-Aldrich, Darmstadt, Germany). The cell confluence in each well was monitored continuously every 6 h using an IncuCyte S3 Live Cell Analysis System (Sartorius, Gottingen, Germany) until at least 72 h.

### 2.9. Cell Viability Assay 

The cell viability was quantified using the water-soluble tetrasolium salt (WST-1) colorimetric assay (Sigma-Aldrich, Darmstadt, Germany). The GIST 882 or 882R, GIST T1 or T1R, and GIST 48 cells were seeded for 24 h at densities of 20,000, 10,000, and 20,000 cells/well in 96-well plates. Then, the cells were treated with 100 µM of 3-BP for 48 h. After treatment, a WST-1 reagent (10 μL) was added to each well for 4 h at 37  °C. The absorbance was measured at 450 nm.

### 2.10. Giemsa Staining of Adherent Cells

The GIST cells were seeded in a 24-well plate until full confluence. After treatment with 100 µM of 3-BP for 8 h, the non-adherent cells were removed by rinsing gently with PBS twice. The remaining adherent cells were fixed with methanol and stained with Giemsa staining solution (Sigma-Aldrich, Darmstadt, Germany).

### 2.11. Annexin V Apoptosis Assay

After treatment with DMSO, 100 µM 3-BP (8 h) or 10 µM gossypol (24 h), both the floating and adherent cells were collected and stained with Annexin V-APC and propidium iodide (Thermo Fisher Scientific, Waltham, MA, USA) for 30 min at room temperature. The percentage of apoptotic cells was determined using NovoCyte Flow Cytometer (ACEA Biosciences, San Diego, CA, USA).

### 2.12. Western Blot Analysis

The cells were lysed using the NP-40 lysis buffer regimen, as previously described [14]. Proteins were quantified with the bicinchoninic acid method (Thermo Fisher Scientific, Waltham, MA, USA). The total protein (30–50 µg) was separated by 4%–12% Bis-Tris NuPAGE gel with a 3-(N-morpholino)propanesulfonic acid (MOPS) buffer and transferred to a nitrocellulose membrane. The membranes were blocked with 5% bovine serum albumin (Sigma-Aldrich). The membranes were incubated at 4 °C overnight with the primary antibodies in 3% bovine serum albumin against phosphorylated AKT at Ser473 (pAKT; dilution 1:1000, Cell Signaling, #9271), AKT (1:1000, Cell Signaling, #9272), PGC1α (1:500, Abcam, #ab77210), mitochondrial transcription factor A (TFAM) (1:1000, Cell Signaling, #8076), lactate dehydrogenase A (LDHA) (1:1000, Cell Signaling, #3582), LDHB (1:1000, Abnova, #H00003945-M01), total OXPHOS (1:200, Abcam, #110413), Glyceraldehyde 3-phosphate dehydrogenase (GAPDH) (1:1000, Cell Signaling, #5174), β-actin (1:2000, Sigma-Aldrich, #A2228), Hexokinase 2 (HK2) (1:1000, Abcam, #ab104836), Hexokinase 1 (HK1) (1:1000, Cell signaling, #2024), Nuclear Respiratory Factor 1 (NRF1) (1:1000, Cell signaling, #46743), nuclear respiratory factor 2 (NRF2) (1:1000, Cell signaling, #12721), and poly (ADP-ribose) polymerase (PARP) (1:1000, BD Biosciences, #556362). IRDye 800CW anti-mouse or 680RD anti-rabbit (1:10,000; LI-COR Biosciences, Lincoln, NE, USA) was used as the secondary antibody. The blots were scanned using an Odyssey LI-COR scanner and analyzed with LI-COR Image Studio software (LI-COR Biotechnology, Lincoln, NE, USA).

### 2.13. Statistical Analysis

All the in vitro experiments were evaluated with at least three independent biological replicates. Statistical analyses were performed using GraphPad Prism 8 (GraphPad Software, San Diego, CA, USA). All the data are expressed as means ± standard deviations. The experiments were analyzed with a two-sided Student’s *t*-test for two groups. For comparisons between more than two groups, a one-way analysis of variance (ANOVA) was used. A two-way ANOVA was applied for more than two groups of two variables, followed by a post hoc test (Dunnett’s). Mann–Whitney U-test was used to compare the protein expressions in the clinical samples. Significance is indicated as *** for a *p* < 0.001, ** for a *p* < 0.01, and * for a *p* < 0.05.

## 3. Results

We first established imatinib-resistant derivative cell lines named GIST 882R (from GIST 882) and GIST T1R (from GIST T1) by incubating with 1 μM of imatinib for 8–10 months. The half maximal inhibitory concentration (IC50) of GIST 882, T1, 882R, and T1R was 0.05 μM, 0.01 μM, 5.09 μM, and 4.89 μM, respectively (Figure S1a). The two cell lines with acquired resistance to imatinib (GIST 882R and T1R) had a comparable IC50 with GIST 48 (4.90 μM, Figure S1a) and sustained cell growth upon 1 μM of imatinib treatment (Figure S1b,c). 

### 3.1. Characterization of OXPHOS and Mitochondrial Biogenesis upon Imatinib Treatment

**Figure 1:**
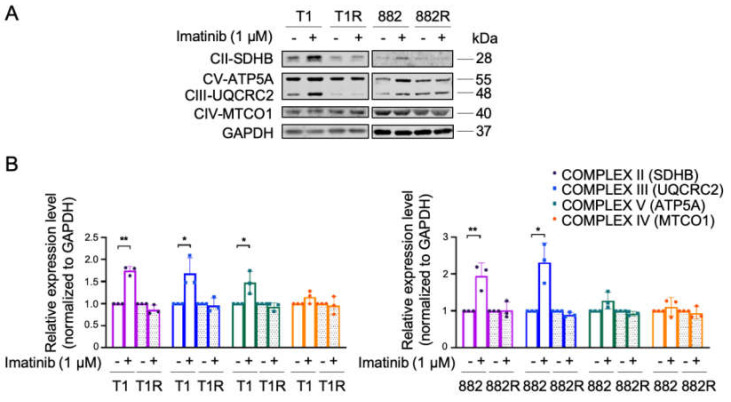
Expressions of glycolysis and oxidative phosphorylation (OXPHOS) proteins in imatinib-sensitive and imatinib-resistant gastrointestional stromal tumors (GIST) cell lines treated with or without 1 µM of imatinib for 48 h. (**A**) Representative immunoblots of OXPHOS proteins are shown. Estimated protein sizes are given to the right in kiloDalton (kDa). (**B**) The quantification of OXPHOS protein expression levels was analyzed from 3 independent biological experiments. Glyceraldehyde 3-phosphate dehydrogenase (GAPDH) was used as an endogenous loading control. Histograms represent mean ± SD. * *p* < 0.05, ** *p* < 0.01 (Student’s *t*-test).

We examined the effect of imatinib on the mitochondrial respiratory function and mitochondrial biogenesis. Expressions of several OXPHOS proteins were increased in both GIST T1 and 882, while no significant alteration of OXPHOS proteins was observed in GIST T1R and 882R (Figure 1a,b). An increased mitochondrial capacity was also observed in the imatinib-sensitive GIST T1 cell line but not in 882 upon imatinib treatment, which is consistent with previous results reported by Vitiello et al. [11]; however, no effect was observed in the imatinib-resistant cell lines (Figure S2). These results suggest that OXPHOS levels are upregulated in imatinib-sensitive GIST as a metabolic adaptation to imatinib, confirming the findings by Vitiello et al. [11]. On the other hand, this metabolic switch secondary to imatinib was not observed in imatinib-resistant GIST.

**Figure 2:**
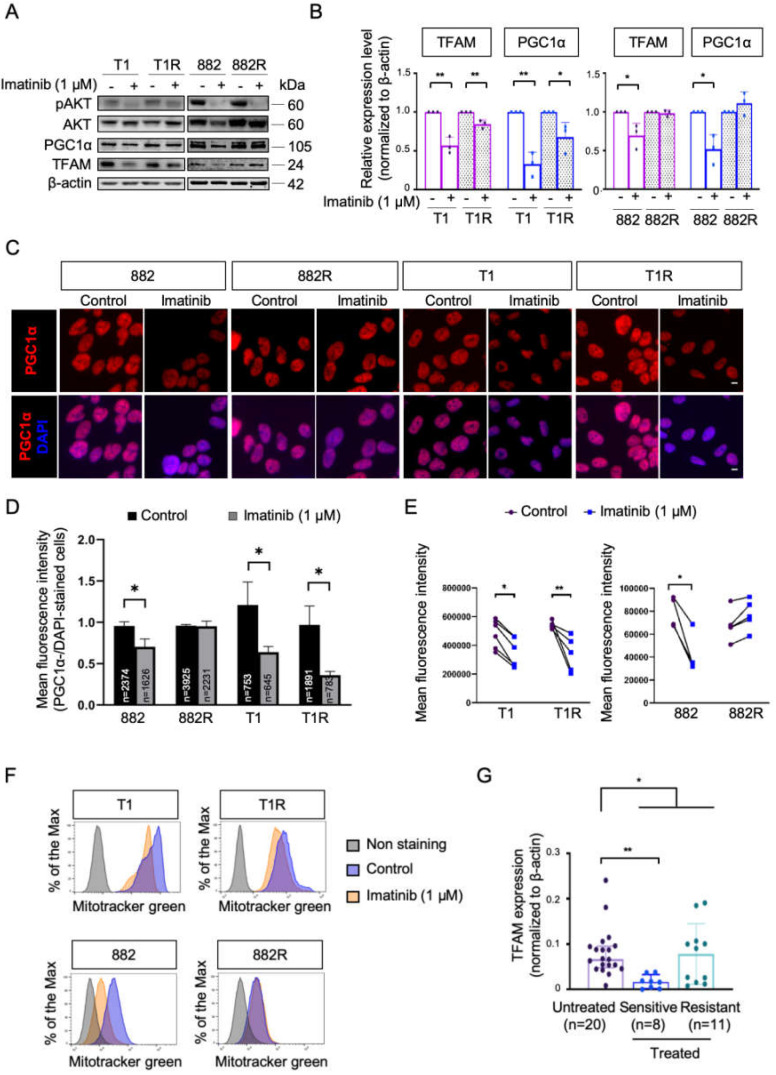
The effect of imatinib on mitochondrial biogenesis in imatinib-sensitive and -resistant GIST cell lines and tumors. (**A**) Representative immunoblots of phosphorylated AKT (pAKT), AKT, peroxisome proliferator-activated receptor coactivator-1 alpha (PGC1α), and mitochondrial transcription factor A (TFAM) are shown. The efficacy of imatinib treatment was demonstrated by a marked reduction in pAKT level. (**B**) Quantification of protein expression of PGC1α and TFAM was normalized to β-actin from 3 independent biological experiments. Histograms represent mean ± SD. * *p* < 0.05, ** *p* < 0.01 (Student’s *t*-test). (**C**) Representative immunofluorescence images show staining for PGC1α (red) and 4′,6-diamidino-2-phenylindole (DAPI) (blue) at 20× objective lens. Scale bar: 10 μm. (**D**) Quantification of PGC1α fluorescence intensity normalized to DAPI-stained nuclei in three different fields. The total number of cells analyzed in each condition is highlighted in the bar graph. * *p* < 0.05 (Student’s *t*-test). (**E**) Dots represent the mean fluorescence intensity of MitoTracker Green detected by flow cytometry, indicating the total mitochondrial mass from at least 4 independent experiments. * *p* < 0.05, ** *p* < 0.01 (Student’s *t*-test). (**F**) Representative examples of MitoTracker Green are shown in histograms. (**G**) Protein expressions of TFAM in the GIST tumor samples were analyzed by western blotting. * *p* < 0.05, ** *p* < 0.01 (Mann–Whitney U-test).

Given the varied responses of OXPHOS between the imatinib-sensitive and -resistant GIST cells, we further investigated the effect of imatinib on the expression of two key activators of mitochondrial biogenesis which are known to drive OXPHOS activity [15]. A decrease in mitochondrial transcription factor A (TFAM) and peroxisome proliferator-activated receptor coactivator-1 alpha (PGC1α) were found in imatinib-sensitive GIST cells upon imatinib treatment (Figure 2a,b). Similarly, the nuclear respiratory factor 2 (NRF2), a transcription factor of TFAM, was also downregulated in imatinib-sensitive GIST cell lines in response to imatinib treatment (Figure S3). Imatinib also reduced mitochondrial biogenesis in GIST T1R, however the effect was subtle. No significant alteration of TFAM, NRFs, and PGC1α in response to imatinib was found in GIST 882R (Figure 2a,b, Figure S3). Moreover, immunofluorescence assays also showed a decreased expression of PGC1α in GIST 882, T1, and T1R cells treated with 1 μM of imatinib, whereas no obvious change was noted in the GIST 882R cells (Figure 2c,d). These results were in line with the alterations in MitoTracker Green as an indicator of mitochondrial mass (Figure 2e,f). Specifically, imatinib reduced the fluorescence intensity of MitoTracker in sensitive GIST T1 and 882 cells and resistant T1R cells. However, no significant change was observed in the resistant 882R cells. Furthermore, the TFAM expression levels in imatinib-sensitive tumor samples (*n* = 8) were lower than in samples from untreated patients (*n* = 20), while we did not find a statistically significant difference between untreated and imatinib-resistant (*n* = 11) cases (Figure 2g). These results indicate that imatinib reduced mitochondrial metabolism in imatinib-sensitive GIST cells. However, the diverse responses of mitochondria biogenesis suggest varied metabolic phenotypes in imatinib-resistant GIST.

### 3.2. Heterogeneity of Energy Metabolism among Imatinib-Resistant GIST Cells

**Figure 3:**
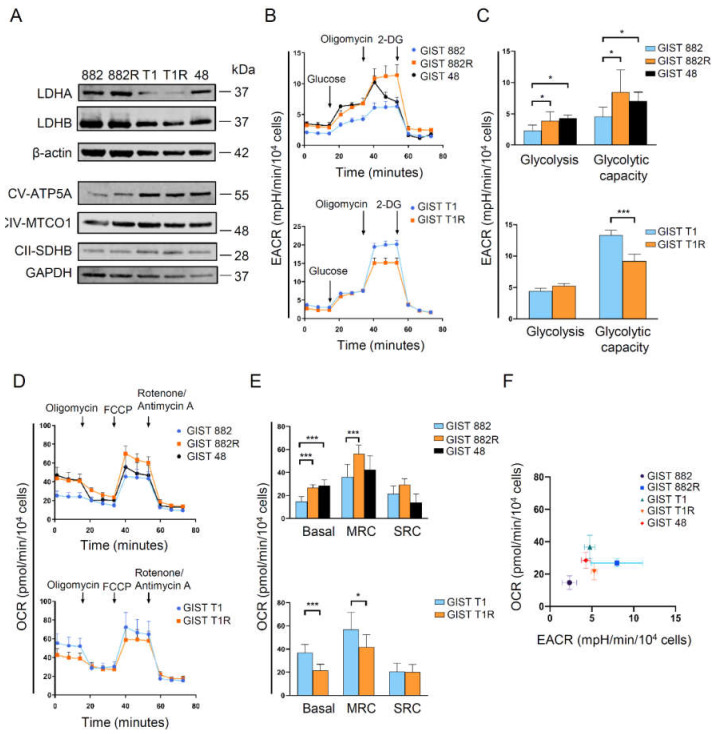
Bioenergetic phenotypes of imatinib-sensitive and -resistant GIST cell lines. (**A**) Basal expression levels of lactate dehydrogenases A and B (LDHA, LDHB) and OXPHOS were detected by immunoblotting. (**B**) The average extra cellular acidification rate (EACR) was measured by the glycolytic stress assay. 2-DG, 2-deoxyglucose. (**C**) The quantification of glycolysis and glycolytic capacity was analyzed by calculating the average EACR between glucose and oligomycin injection and between oligomycin and 2-DG injection, respectively. Histograms represent mean ± SD. * *p* < 0.05, *** *p* < 0.001 (Student’s *t*-test, *n* = 2). (**D**) The average oxygen consumption rate (OCR) was measured by the mitochondrial stress assay. FCCP, carbonyl cyanide-4 (trifluoromethoxy) phenylhydrazone. (**E**) Basal OCR, maximal respiratory capacity (MRC), and spare respiratory capacity (SRC) were quantified to evaluate the mitochondrial respiratory function under basal and stressed conditions. Histograms represent mean ± SD. * *p* < 0.05, *** *p* < 0.001 (Student’s *t*-test, *n* = 2) (**F**) The energy map was plotted according to the basal OCR and EACR. Data represent mean ± SD.

Lactate dehydrogenases A and B (LDHA and LDHB, respectively) are two common subunits of LDH that promote interconversion between pyruvate and lactate. More specifically, LDHA catalyzes the reduction of pyruvate to lactate for glycolysis, and LDHB converts lactate to pyruvate for OXPHOS [16]. We found that GIST 882R cells have a higher glucose uptake and protein expressions of LDHA and OXPHOS than GIST 882 cells (Figure 3a, Figure S4), suggesting that a subset of imatinib-resistant GIST cells develop a phenotype with a high metabolic activity. On the other hand, no difference in LDH and OXPHOS protein expressions were observed between the GIST T1R and T1 cells. Since imatinib has been reported to decrease the glycolysis capacity in GIST [11], we also evaluated whether imatinib altered the expressions of LDHA and LDHB. However, we did not find significant changes in LDHA or LDHB expression upon imatinib treatment in both sensitive and resistant GIST cell lines (Figure S3). To determine the bioenergetic phenotype of parental GIST cells and their daughter cells acquiring imatinib resistance, we measured the baseline and stressed conditions of glycolysis and mitochondria respiration using the Seahorse system. The increase in the extracellular acidification rate (EACR) after adding glucose was more pronounced in GIST 882R than 882 cells, indicating that the basal glycolysis was elevated in the GIST 882R cells (Figure 3b,c). The glycolytic capacity was also higher in GIST 882R than 882 cells. The basal mitochondrial respiration and maximal respiratory capacity, measured as the oxygen consumption rate (OCR), were increased in GIST 882R as compared to 882 cells (Figure 3d,e). Similarly, imatinib-resistant GIST 48 cells also manifested a higher basal EACR and OCR than GIST 882 cells. These findings indicate a highly metabolically active phenotype in subsets of resistant GIST cells (Figure 3f). In contrast, GIST T1R cells exert a similar basal EACR and lower basal OCR to GIST T1 cells (Figure 3b–e), suggesting that a subset of imatinib-resistant GIST manifests a low OXPHOS phenotype (Figure 3f). During OXPHOS, reactive oxygen species (ROS) are generated by mitochondria [17]. We found decreased intracellular ROS levels in both resistant cell lines as compared to their sensitive parental cells (Figure S5), which is concordant with a low ROS production in imatinib-resistant chronic myeloid leukemia cell lines [18]. 

### 3.3. Cytotoxic Responses to Inhibition of Glycolysis and Oxidative Phosphorylation 

**Figure 4:**
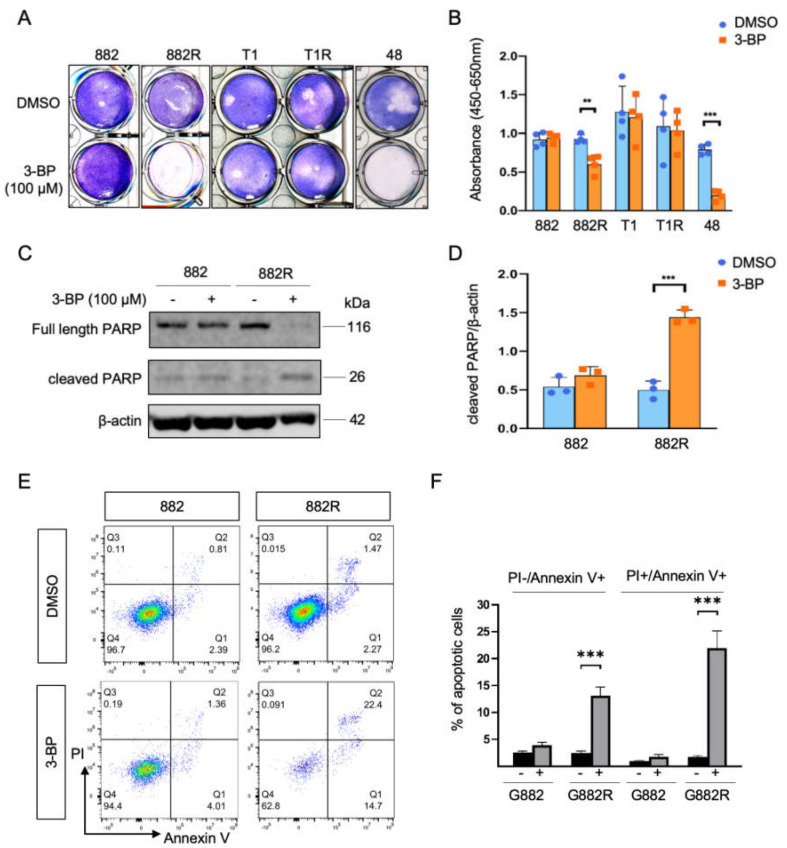
Cytotoxic effect of 3-bromopyruvate (3-BP) on GIST cells (**A**) Giemsa staining shows the number of adherent cells upon treatment with 3-BP (100 µM, 8 h) or dimethyl sulfoxide (DMSO). (**B**) The viability of GIST cells treated with 3-BP was measured by the WST-1 assay. Histograms represent mean ± SD. ** *p* < 0.01, *** *p* < 0.001 (Student’s *t*-test). (**C**) Representative immunoblots of full length and cleaved poly (ADP-ribose) polymerase (PARP) in the GIST 882 and 882R cells treated with 3-BP or DMSO. (**D**) Quantification of cleaved PARP/β-actin ratio from three independent biological experiments. (**E**) Representative flow cytometric images of the effect of 3-BP treatment on cell apoptosis by AnnexinV-APC and propidium iodide (PI) staining. The early and late apoptotic cells are presented by Annexin V+/PI− (lower right quadrant, Q1) and Annexin V+/PI+ (upper right quadrant, Q2) stained cells, respectively. (**F**) Quantification of the apoptotic cells from three independent biological experiments. Histograms represent mean ± SD. *** *p* < 0.001, (Student’s *t*-test).

We further evaluated whether imatinib-resistant GIST cells are more metabolically vulnerable than imatinib-sensitive GIST cells. We observed that inhibition of glycolysis by adding 100 µM of 3-BP, an inhibitor of hexokinase 2 (HK2), increased loosely adherent/floating cells in GIST 882R and GIST 48, while the GIST 882, T1, and T1R cells were not affected at the same concentration (Figure 4a). A significant decrease in viability was also observed in GIST 882R and 48 cells using a WST-1 assay (Figure 4b). Cleaved poly (ADP-ribose) polymerase (PARP) is an indicator of apoptosis. The upregulated protein expression of cleaved PARP was found in GIST 882R cells treated with 3-BP (100 µM, 8 h; Figure 4c,d). Consistent with the cleaved PARP level, the 3-BP treatment increased both the early and late apoptotic cells, as evaluated by the annexin V and propidium iodide assay (Figure 4e,f). Noteworthy, 3-BP only reduced the HK2, but not HK1, levels in GIST 882R and had no effect on the 882 cells (Figure S6), indicating the specificity of the inhibitor in the suppression of HK2 that led to apoptosis in the 882R cells. Together, our results indicate that inhibition of glycolysis reduced cell viability and induced apoptosis.

**Figure 5:**
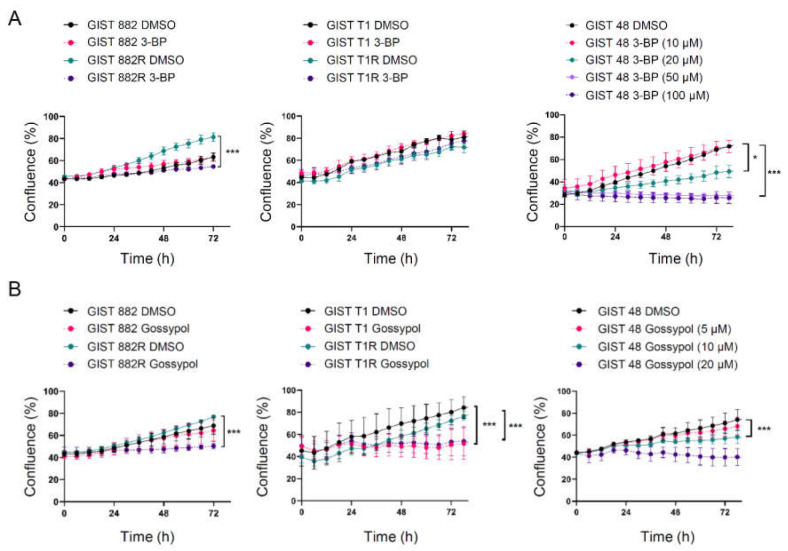
The effect of 3-BP and gossypol on GIST cell proliferation using an Incucyte S3 Live Cell Analysis System. (**A**) The cell confluence (% of occupied area) was measured every 6 h in GIST cells treated with 100 µM of 3-BP or DMSO. GIST 48 cells were treated with 3-BP at various concentrations or DMSO. * *p* < 0.05, *** *p* < 0.001 (Student’s *t*-test or 2-way ANOVA with Dunnett’s test, *n* = 3). (**B**) GIST cells were treated with 10 µM of gossypol or DMSO. GIST 48 cells were treated with DMSO or various concentrations of gossypol. *** *p* < 0.001 (Student’s *t*-test or 2-way ANOVA with Dunnett’s test, *n* = 3).

Furthermore, the effect of 3-BP on the cell proliferation was also determined using an IncuCyte live-cell imaging system. The GIST 882R cells were more vulnerable to 3-BP than GIST 882 cells at the same concentration of 100 µM (Figure 5a, left panel). The GIST T1 and T1R cells had similar proliferation rates when exposed to 3-BP (Figure 5a, middle panel). A significantly reduced proliferation of GIST 48 cells was observed at an even lower concentration (20 µM) of 3-BP (Figure 5a, right panel). Similar findings were observed for GIST 882, 882R, and 48 when using gossypol, which preferentially inhibits glycolysis by suppressing the activity of LDHA (Figure 5b, Figure S7). The gossypol treatment reduced cell growth in both the GIST T1 and T1R cell lines (Figure 5b, middle panel). These results suggest that a subset of imatinib-resistant GIST cells with a high basal glycolysis are subjected to the inhibition of glycolysis.

**Figure 6:**
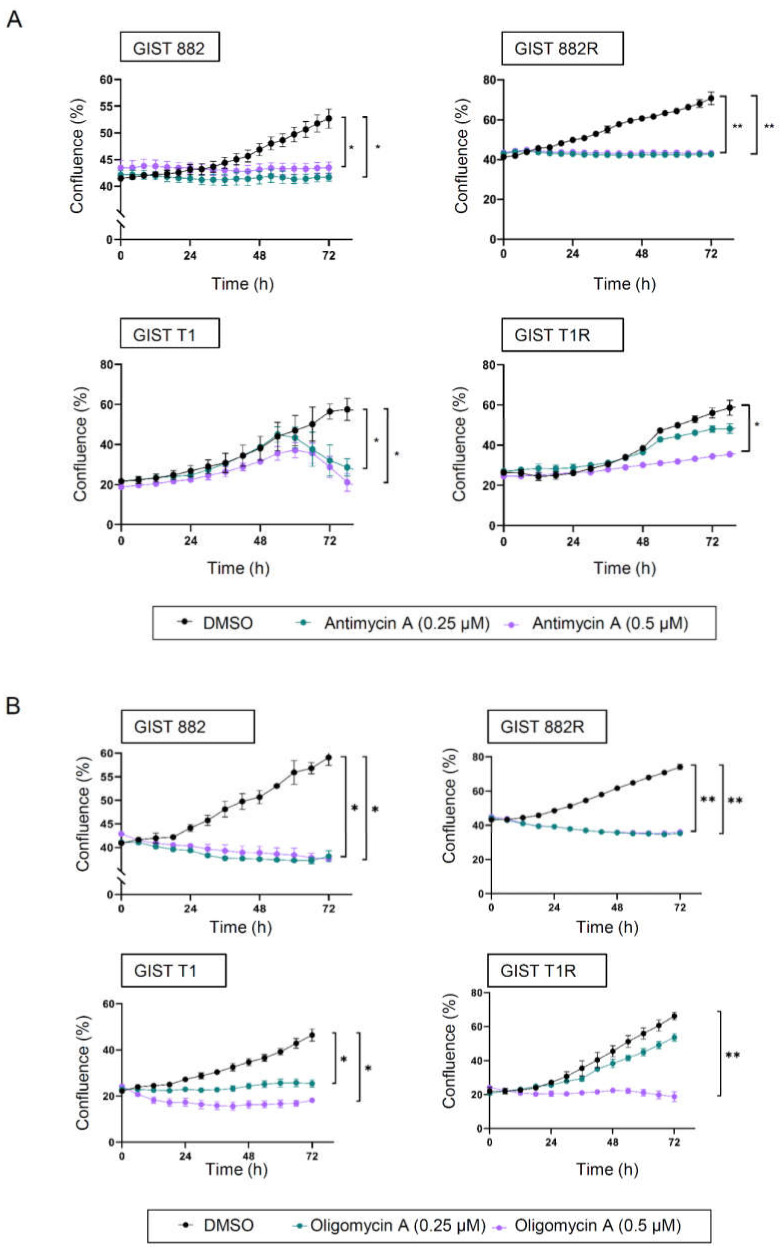
The effect of antimycin A and oligomycin A on GIST cell proliferation. (**A**,**B**) The cell confluence (% of occupied area) was measured every 6 h using an Incucyte S3 Live Cell Analysis System. GIST cells were treated with antimycin A (**A**) or oligomycin A (**B**) at 0.25 µM and 0.5 µM or DMSO. * *p* < 0.05, ** *p* < 0.01 (2-way ANOVA, Dunnett’s test, *n* = 3).

We also assessed whether the inhibition of OXPHOS influences cell growth using antimycin A and oligomycin A, which specifically inhibit complex III and complex V of the electron transport chain, respectively. The cell growth was comparable between the GIST 882 and 882R cells when incubated with the same concentration of antimycin A or oligomycin A (Figure 6a,b). However, only a higher concentration (0.5 µM) of antimycin A or oligomycin A significantly reduced the cell growth of GIST T1R (Figure 6a,b), indicating that these cells were more resistant to both antimycin A and oligomycin A compared to GIST T1.

### 3.4. High Metabolically Active Phenotype in a Subset of Imatinib-Resistant GIST Tumors 

**Figure 7:**
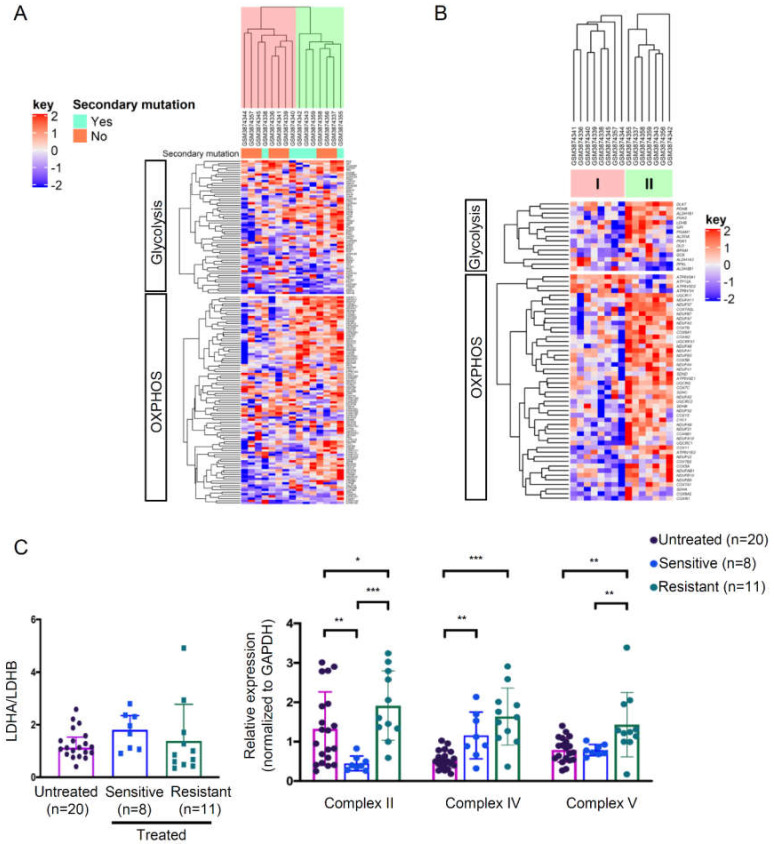
Expressions of OXPHOS and glycolysis genes in the GIST tumor samples. (**A**,**B**) Microarray data were obtained from National Center for Biotechnology Information (NCBI) Gene Expression Omnibus (accession number GSE132542). (**A**) The heatmap represents the gene expressions of all genes involved in the OXPHOS and glycolysis gene sets in imatinib-resistant GIST (*n* = 15). All the samples were clustered using Euclidean distance. The dendrogram indicates two different clusters (highlighted in pink or green) based on the expression levels of OXPHOS and glycolysis gene sets. (**B**) Significantly differentially expressed genes from (**A**) with a false discovery rate of < 0.2 were clustered. (**C**) Protein expression ratios between LDHA and LDHB and Complex II, IV, and V in GIST tumors (*n* = 39) were analyzed by western blotting. Histograms represent mean ± SD. * *p* < 0.05, ** *p* < 0.01, *** *p* < 0.001 (Mann–Whitney U-test).

To investigate the energy metabolism of imatinib-resistant GIST tumors, we analyzed a Gene Expression Omnibus (GEO) dataset which included 15 imatinib-resistant tumors [19]. The heatmap presented in Figure 7a illustrates the expression levels of all genes in the two selected gene sets for OXPHOS (*n* = 95) and glycolysis (*n* = 61) (Table S3). The samples were grouped into two clusters based on the gene expression pattern. Based on the fold changes between cluster II and cluster I, we identified 49 differentially expressed genes (false discovery rate of <0.2) involved in the regulation of OXPHOS and 15 in glycolysis (Figure 7b; Table S3). Generally, cluster II displayed higher expressions of both glycolysis and OXPHOS genes, indicating a highly metabolically active phenotype. On the other hand, cluster I showed a relatively low bioenergetic status. These results suggest a heterogenous energy metabolism in imatinib-resistant GISTs, which is in line with the bioenergetic profiling from the GIST cell lines. In addition, we evaluated the expressions of key proteins regulating glycolysis and OXPHOS in our GIST cohort. We did not find statistically significant differences in the LDHA/LDHB ratio between the imatinib-resistant or -sensitive samples and the untreated samples (Figure 7c, Figure S8). However, the OXPHOS expressions of the imatinib-resistant samples were significantly increased compared with the untreated samples (for Complexes II, IV, and V) or imatinib–sensitive samples (for Complexes II and V) (Figure 7c, Figure S9).

## 4. Discussion

In this study, we characterized the bioenergetic profiles of imatinib-sensitive and -resistant GIST cell lines and the relationships between bioenergetic features and sensitivity to metabolic inhibitors. We showed that GIST 882R cells with a high metabolically active phenotype were susceptible to glycolysis and OXPHOS inhibitions, whereas GIST T1R cells, with a low OXPHOS phenotype, were resistant to 3-BP and only sensitive to antimycin A or oligomycin A with a higher dosage. The gene expression profiling revealed two distinct subgroups of imatinib-resistant tumors based on the glycolysis and OXPHOS gene sets. These results demonstrate metabolic vulnerability in a subset of resistant GIST, which provides new insight for overcoming resistance to KIT inhibitor.

PGC1α is the master regulator of mitochondrial biogenesis through the NRF1/2-mediated regulation of TFAM, which drives the replication and transcription of mitochondrial DNA, including the synthesis of OXPHOS subunits [15]. Here, we observed that imatinib induced OXPHOS but decreased PGC1α, TFAM, NRF2, and mitochondrial mass in imatinib-sensitive GIST cells, suggesting that imatinib suppresses not only glycolysis but also PGC1α-dependent mitochondrial biogenesis. On the other hand, we observed diverse PGC1α responses to imatinib in resistant GIST cells. For GIST 882R cells, we did not observe any changes in PGC1α level and mitochondrial biogenesis, suggesting that this resistant cell line is tolerant to imatinib stress without switching bioenergetic adaptation. For GIST T1R, we observed decreased PGC1α and TFAM levels as well as mitochondrial mass, which was similar to the effect observed in the parental GIST T1.

Generally, the OXPHOS level is positively correlated with mitochondrial biogenesis. However, a negative correlation between OXPHOS and mitochondrial biogenesis has also been found. For example, CD8 T lymphocytes have impaired mitochondrial responses during virus infection, resulting in increased mitochondrial mass but reduced OXPHOS [20,21]. On the other hand, upregulated OXPHOS without the alteration of mitochondrial mass could be attributed to mitochondrial fusion [22] or highly active mitochondria [23].

Cancer cells generally utilize glycolysis for ATP production instead of mitochondrial respiration even in the presence of adequate oxygen, also known as the Warburg effect [24]. Chemo-resistance or resistance to oncogene addiction has been shown to shift from glycolysis toward OXPHOS [4,25,26,27,28]. Glycolysis inhibition and increased ATP demand under the stress of anticancer agents may, at least in part, explain the metabolic rewiring of resistant cells. In support of this hypothesis, we found an increased expression of OXPHOS proteins in imatinib-resistant GIST tumors compared with untreated tumors. Conversely, resistant cells can acquire glycolytic metabolism for surviving cytotoxic stress [29,30,31,32]. Similarly, cancer stem cells, which are inherently resistant to therapy, can also adopt glycolysis for energy production depending on MYC expression [33]. These observations indicate the metabolic flexibility of cancer cells that allows them to survive cytotoxic agents. Concordantly, imatinib-resistant GIST 882R and 48 cell lines have a high basal glycolytic activity and they were more vulnerable to glycolysis inhibition. The observation of high glycolytic activity in cancer cells with acquired resistance to imatinib is in agreement with the clinical finding of the re-emergence of metabolic activity following a period of imatinib treatment measured by fluorine-18-fluorodeoxyglucose uptake [34].

Interestingly, GIST 882R cells exhibit a high metabolically active phenotype with both high glycolysis and OXPHOS, which is in contrast to the parental GIST 882 cells. This bioenergetic phenotype has been observed in other cancer types [35,36]. Cancer cells with a relatively high metabolically active phenotype may provide more flexibility to survive than those with either a glycolytic or oxidative phenotype. In contrast, GIST T1R cells exhibit a low OXPHOS type compared with the parental GIST T1 cells. The growth of GIST T1R cells was not effectively inhibited by 3-BP and less sensitive to antimycin A or oligomycin A compared to the parental GIST T1 cells, suggesting that a subset of imatinib-resistant GIST cells may not upregulate mitochondrial metabolism to maintain cell growth. Notably, the effect of the two glycolysis inhibitors was different for both GIST T1 and T1R. Both cell lines were sensitive to gossypol but not to 3-BP. Although gossypol has been widely described as a LDHA inhibitor [37], it has also been shown to affect OXPHOS [38,39] and bind directly to the BH3 binding pocket of the Bcl-2 family [40,41]. The effect of gossypol treatment in GIST T1 and T1 R could be due to the combination of glycolysis inhibition and/or other activities exerted by gossypol. The mechanism of the gossypol-mediated growth inhibition of these cells has yet to be determined. Despite that 3-BP, oligomycin A, and antimycin A have been widely used as specific metabolic inhibitors; we have not completely excluded the effect of these inhibitors on other signaling pathways. For 3-BP, although we (in this study) and others have demonstrated its effect on HK2 expression, whether 3-BP has effects on other HK isoenzymes, such as HK3 and HK4, also warrants further studies.

Together, our results suggest the existence of the inter-tumor heterogeneity of metabolic phenotype in imatinib-resistant GIST. In line with our findings, metabolic heterogeneity among drug resistant cells has been reported in several cancer types, including breast, lung, pancreatic, and ovarian cancers [28,35,36,42].

## 5. Conclusions

We highlight the bioenergetic heterogeneity of imatinib-resistant GIST cell lines and tumor samples. Resistant cells can develop a relatively higher metabolically active phenotype than the parental sensitive cells, whereas a subset of resistant cells may exhibit a low OXPHOS status. We also demonstrate the sensitivity of metabolic inhibitors related to varied bioenergetic properties, providing a rationale for targeting energy metabolism to overcome imatinib resistance. 

## Supplementary Materials

The following are available online at https://www.mdpi.com/2073-4409/9/6/1333/s1: Figure S1: Cytotoxicity effect of imatinib on GIST cell lines. (A) GIST cell lines were treated with imatinib at various concentrations for 72 h. The half maximal inhibitory concentration (IC_50_) was determined by WST-1 assay. (B) Growth confluency upon treatment with 1 μM imatinib was measured in real-time up to 96 h using an Incucyte S3 Live Cell Analysis System. (C) Brightfield images show morphological characteristics of GIST cell lines treated with and without 1 μM imatinib. Figure S2. OCR profiles in GIST cell lines with and without 1 µM of imatinib treatment. (A) GIST cell lines were treated with 1 µM of imatinib for 12 h before the mitochondrial stress assay. (B) Quantifications of MRC and SRC relative to their basal levels are shown. Data represent mean ± SD (*n* = 2), * *p* < 0.05 by Student’s *t*-test. Figure S3. Analyses of the effect of imatinib treatment on NRF1, NRF2, LDHA and LDHB expression levels in GIST cells using Western blotting. Representative immunoblots are shown in the upper panel and the quantifications in the low panels. Data represent mean ± SD (*n* = 3), * *p* < 0.05 by Student’s *t*-test. Figure S4. Comparison of glucose uptake in GIST 882 and 882R cells using 2-NBDG assay. Cells were incubated with glucose-depleted medium for 1 h, followed by glucose-free medium containing 2-(*N* -(7-nitrobenz-2-oxa-1,3-diazol-4-yl)amino)-2-deoxyglucose (2-NBDG, 100 µg/mL) for 45 min at 37 °C. After washing, cells containing the dye were detected by flow cytometry at excitation/emission = 485/535 nm. Data represent mean ± SD (*n* = 3), *** *p* < 0.001 by Student’s *t*-test. Figure S5. Quantification of reactive oxygen species (ROS) in GIST cell lines. Intracellular ROS levels were measured using 2’,7’-dichlorofluorescin diacetate and flow cytometry. Data represent mean ± SD (*n* = 5). *** *p* < 0.001 by Student’s *t*-test. Figure S6. Detection of human hexokinase enzymes HK1 and HK2 in GIST882 and GIST882R cell lines with and without treatment of 3-BP using Western blot analysis. Representative Western blots are shown in the upper panel and the quantifications of HK1 and HK2 levels are presented in the lower panels. Data represent mean ± SD (*n* = 3). *** *p* < 0.001 Student’s *t*-test. Figure S7. Evaluation of the effect of gossypol treatments on cell apoptosis by AnnexinV-APC and propidium iodide (PI) stainings. Representative flow cytometric images are shown on the left panels and the quantifications are shown on the right panels. The early and late apoptotic cells are presented by Annexin V+/PI- (lower right quadrant, Q1) and Annexin V+/PI+ (upper right quadrant, Q2) stained cells, respectively. Histograms represent mean ± SD. *** *p* < 0.001, (Student’s *t*-test). Figure S8: Immunoblots corresponding to Figure 7C showing LDHA and LDHB in 39 GIST tumors. Imatinib untreated samples are shown at the top and sensitive and resistant imatinib treated samples below. Molecular weight markers (M) are included in all immunoblots and the size markers are given to the right in kiloDalton (kDa). Sample #6-1 was used as a reference for comparison between western blot membranes. Figure S9: Immunoblots corresponding to Figure 7C showing OXPHOS proteins in 39 GIST tumors. Imatinib untreated samples are shown at the top and sensitive and resistant imatinib treated samples below. Molecular weight markers (M) are included in all immunoblots and the size markers are given to the right in kiloDalton (kDa). Sample #6-1 was used as a reference for comparison between western blot membranes. Table S1: Clinical features, mutation status and imatinib treatment for the 35 GIST cases studied. Table S2: Short tandem repeat profiles of the 5 human GIST cell lines in this study. Table **S3:** Differentially expressed genes for OXPHOS and glycolysis gene sets between Cluster I and II.
