# Supplementary material for: Heterogeneity of Metabolic Vulnerability in Imatinib-Resistant Gastrointestinal Stromal Tumor
Source: Cells. 2020 May 26;9(6):1333. doi: 10.3390/cells9061333 (PMC7348861; doi:10.3390/cells9061333)
Supplement: Supplementary file 1 [file cells-09-01333-s001.zip › cells-779087-supplementary/suppl-proof/supplemental figures revised.pdf]

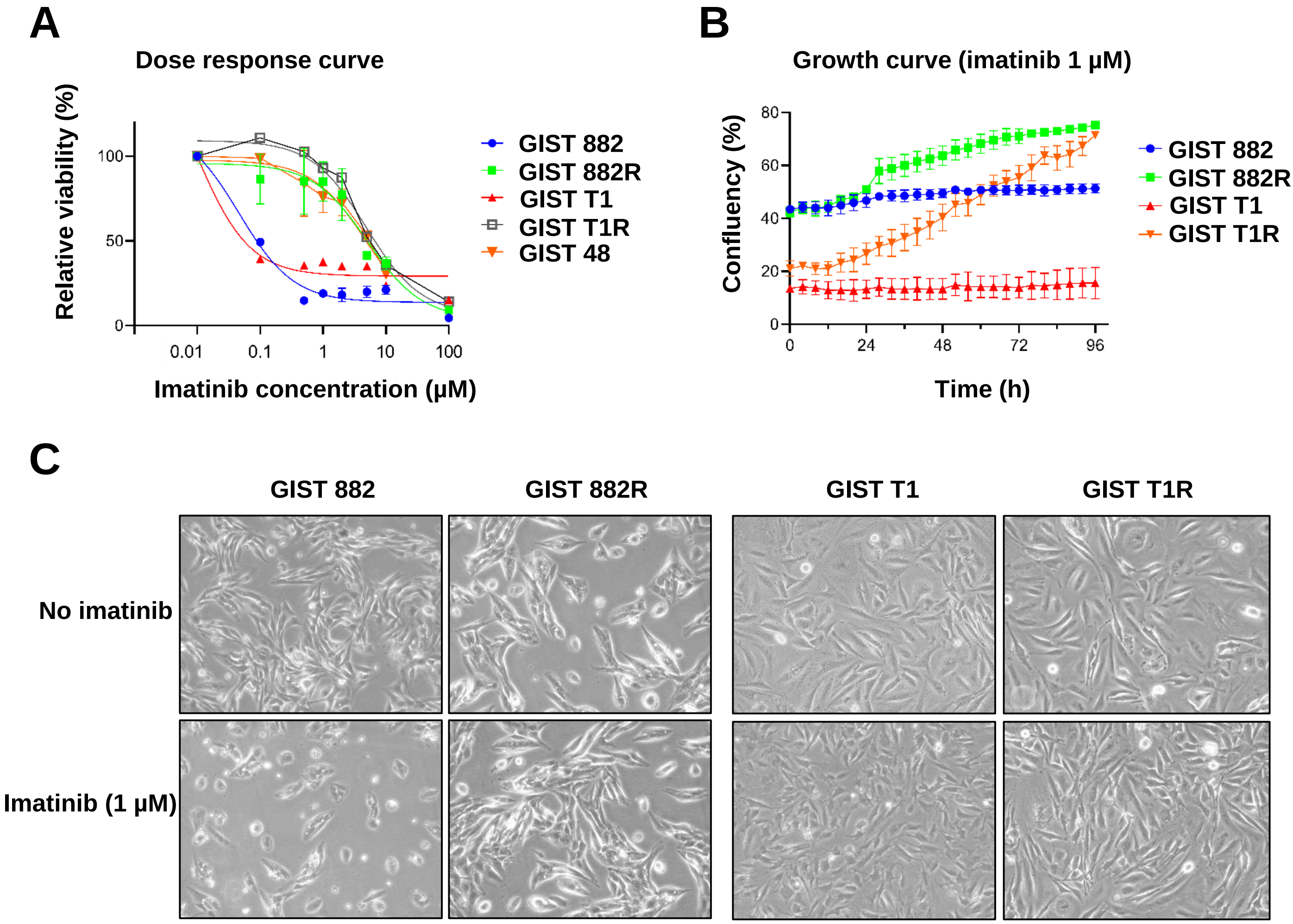

Figure S1. Cytotoxicity effect of imatinib on GIST cell lines. (A) GIST cell lines were treated with imatinib at various concentrations for 72 h. The half maximal inhibitory concentration (IC50) was determined by WST-1 assay. (B) Growth confluency upon treatment with 1 μM imatinib was measured in real-time up to 96 h using an Incucyte S3 Live Cell Analysis System. (C) Brightfield images show morphological characteristics of GIST cell lines treated with and without 1 μM imatinib.

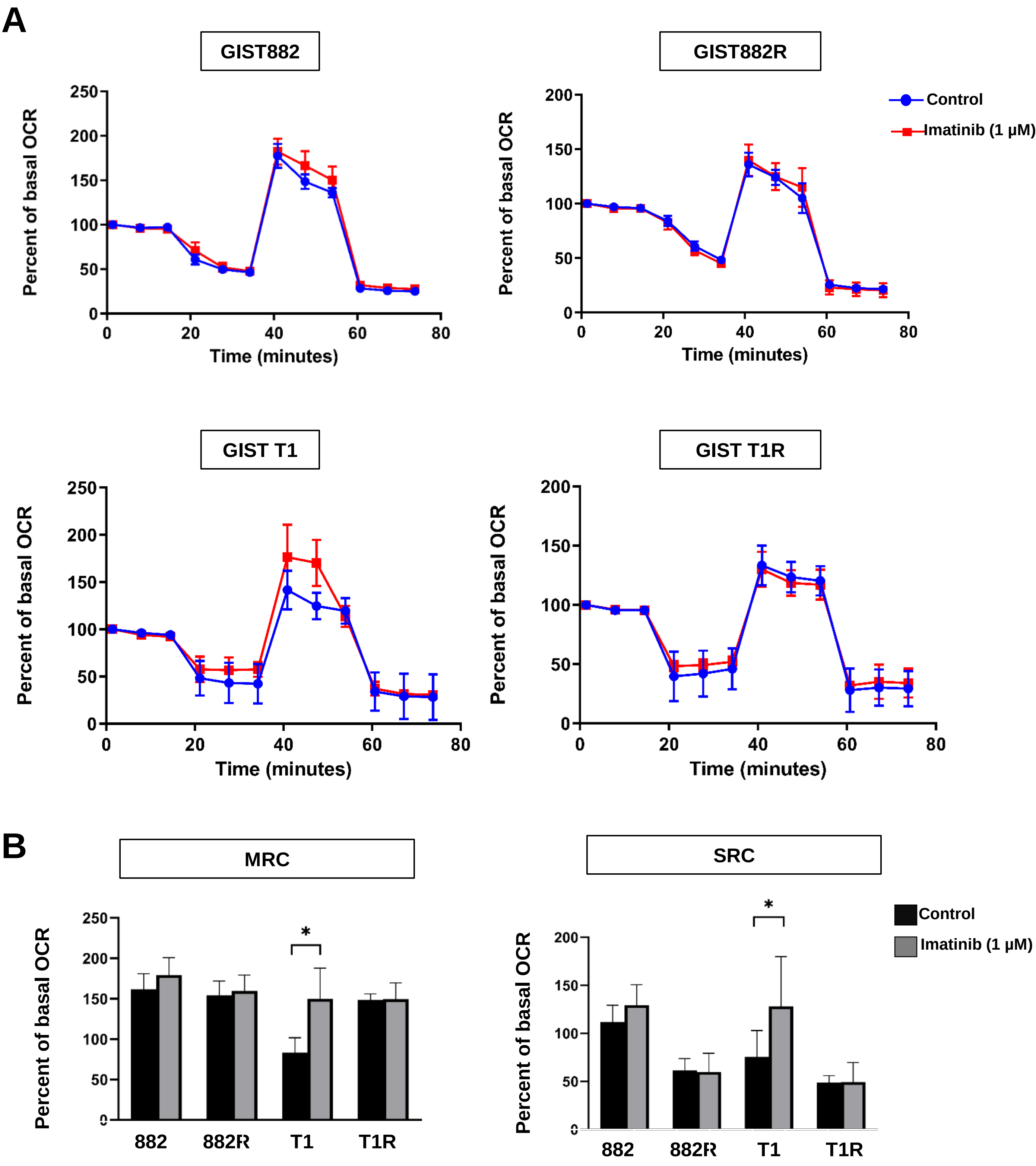

Figure S2. OCR profiles in GIST cell lines with and without 1 μM of imatinib treatment. (A) GIST cell lines were treated with 1 μM of imatinib for 12 h before the mitochondrial stress assay. (B) Quantifications of maximal respiratory capacity (MRC) and spare respiratory capacity (SRC) relative to their basal levels are shown. Data represent mean ± SD (n=2), *$p<0.05$ by Student's t-test.

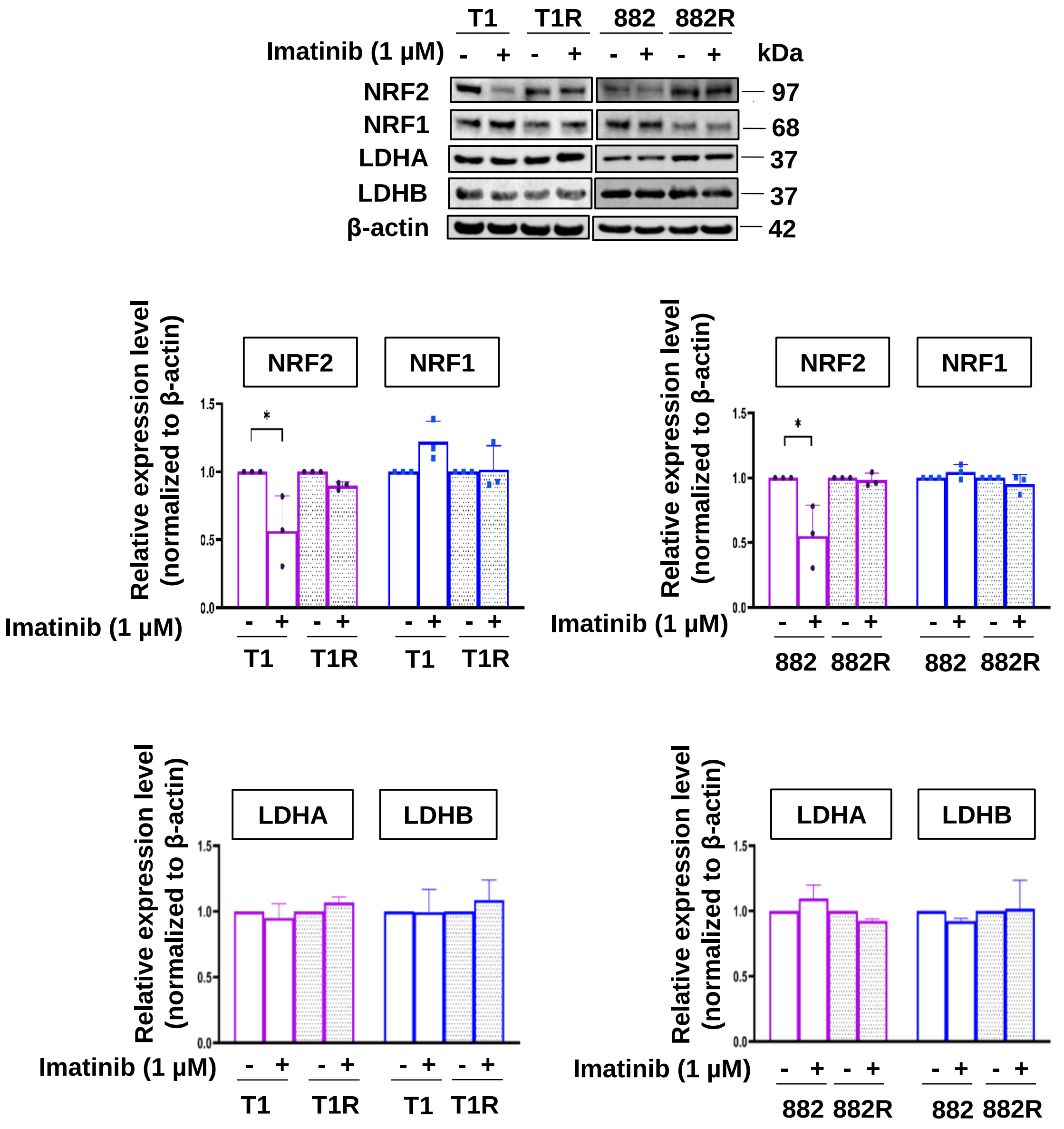

Figure S3. Analyses of the effect of imatinib treatment on NRF1, NRF2, LDHA and LDHB expression levels in GIST cells using Western blotting. Representative immunoblots are shown in the upper panel and the quantifications in the low panels. Data represent mean ± SD (n=3), *p<0.05 by Student's t-test.

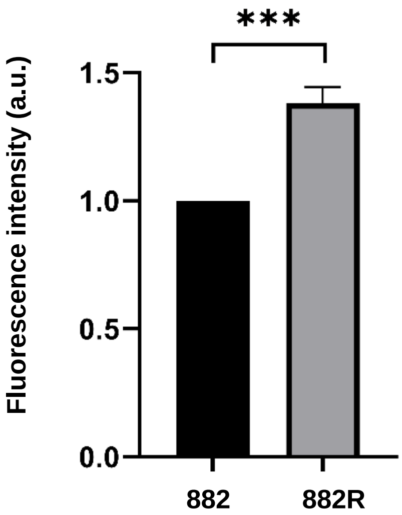

Figure S4. Comparison of glucose uptake in GIST 882 and 882R cells using 2-NBDG assay. Cells were incubated with glucose-depleted medium for 1 h, followed by glucose-free medium containing 2-(N-(7-nitrobenz-2-oxa-1,3-diazol-4-yl)amino)-2-deoxyglucose (2-NBDG, 100 μg/mL) for 45 min at 37 °C. After washing, cells containing the dye were detected by flow cytometry at 485/535 nm wavelengths. Data represent mean ± SD (n=3), ****p*<0.001 by Student's t-test.

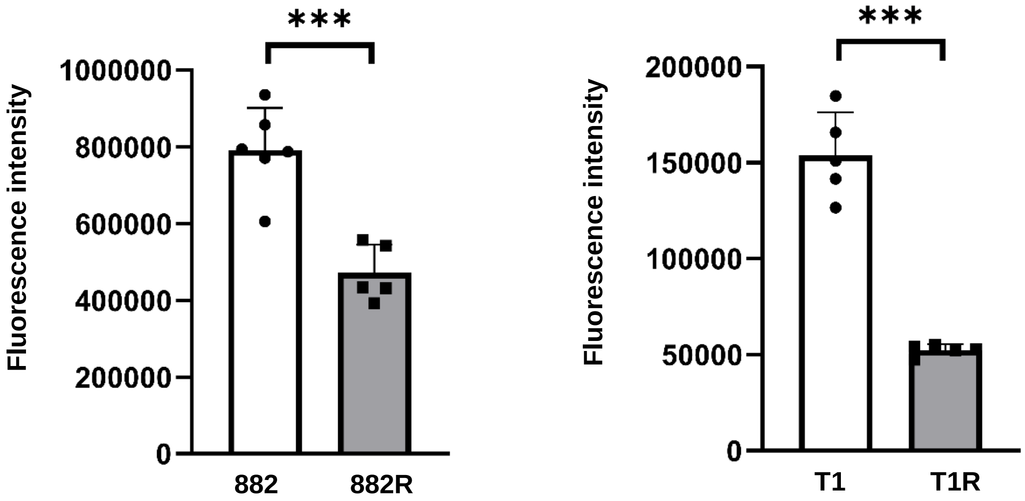

Figure S5. Quantification of reactive oxygen species (ROS) in GIST cell lines. Intracellular ROS levels were measured using 2',7'-dichlorofluorescin diacetate and flow cytometry. Data represent mean ± SD (n=5). ***p<0.001 by Student's t-test.

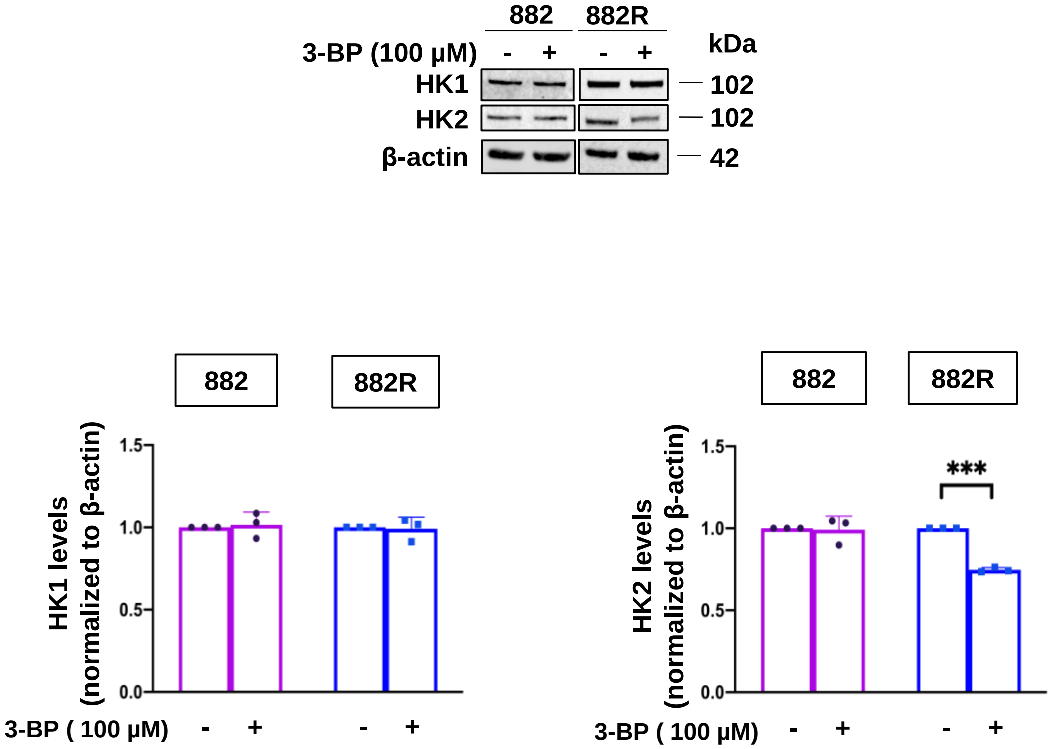

Figure S6. Detection of human hexokinase enzymes HK1 and HK2 in GIST882 and GIST882R cell lines with and without treatment of 3-BP using Western blot analysis. Representative Western blots are shown in the upper panel and the quantifications of HK1 and HK2 levels are presented in the lower panels. Data represent mean ± SD (n=3). ****p*<0.001 by Student's t-test.

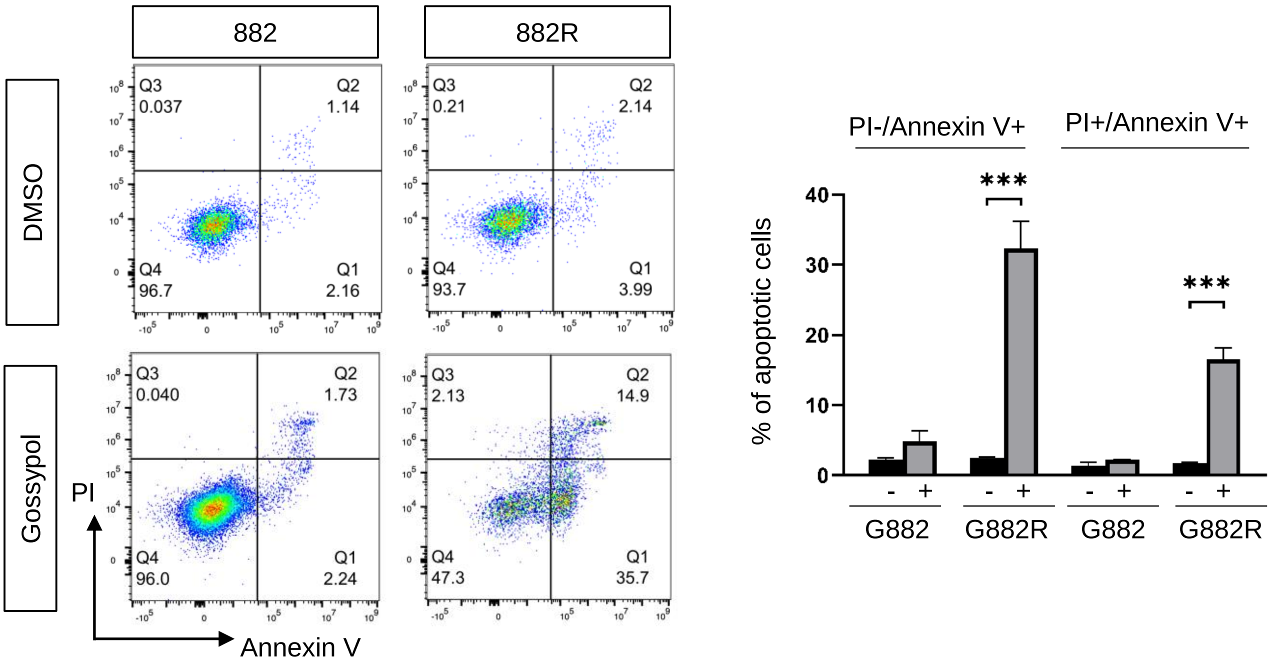

Figure S7. Evaluation of the effect of gossypol treatment (10 μM, 24 h) on cell apoptosis by AnnexinV-APC and propidium iodide (PI) stainings. Representative flow cytometric images are shown on the left panels and the quantifications are shown on the right panels. The early and late apoptotic cells are presented by Annexin V+/PI- (lower right quadrant, Q1) and Annexin V+/PI+ (upper right quadrant, Q2) stained cells, respectively. Histograms represent mean ± SD. ****p*<0.001, (Student's t-test).

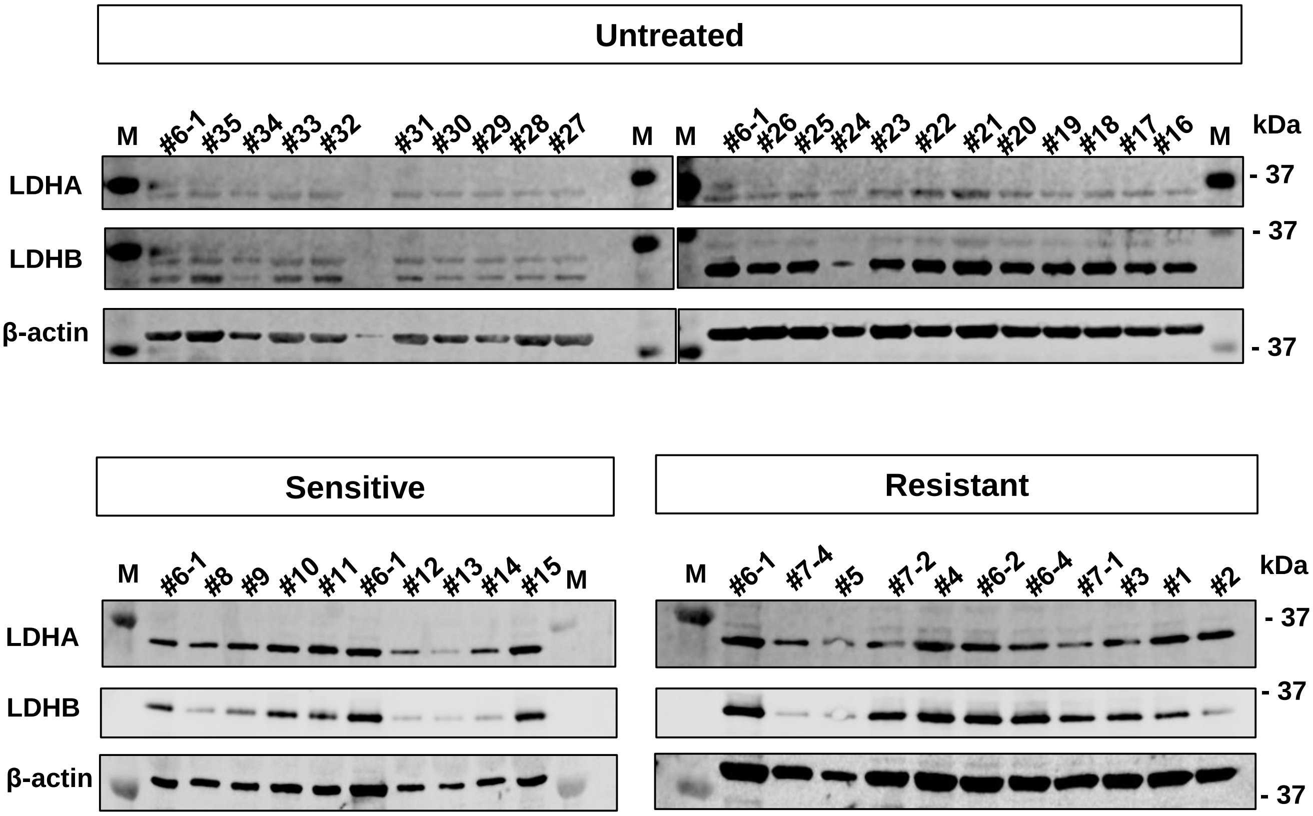

Figure S8. Immunoblots corresponding to Figure 7C showing LDHA and LDHB in 39 GIST tumors. Imatinib untreated samples are shown at the top and sensitive and resistant imatinib treated samples below. Molecular weight markers (M) are included in all immunoblots and the size markers are given to the right in kiloDalton (kDa). Sample #6-1 was used as a reference for comparison between western blot membranes.

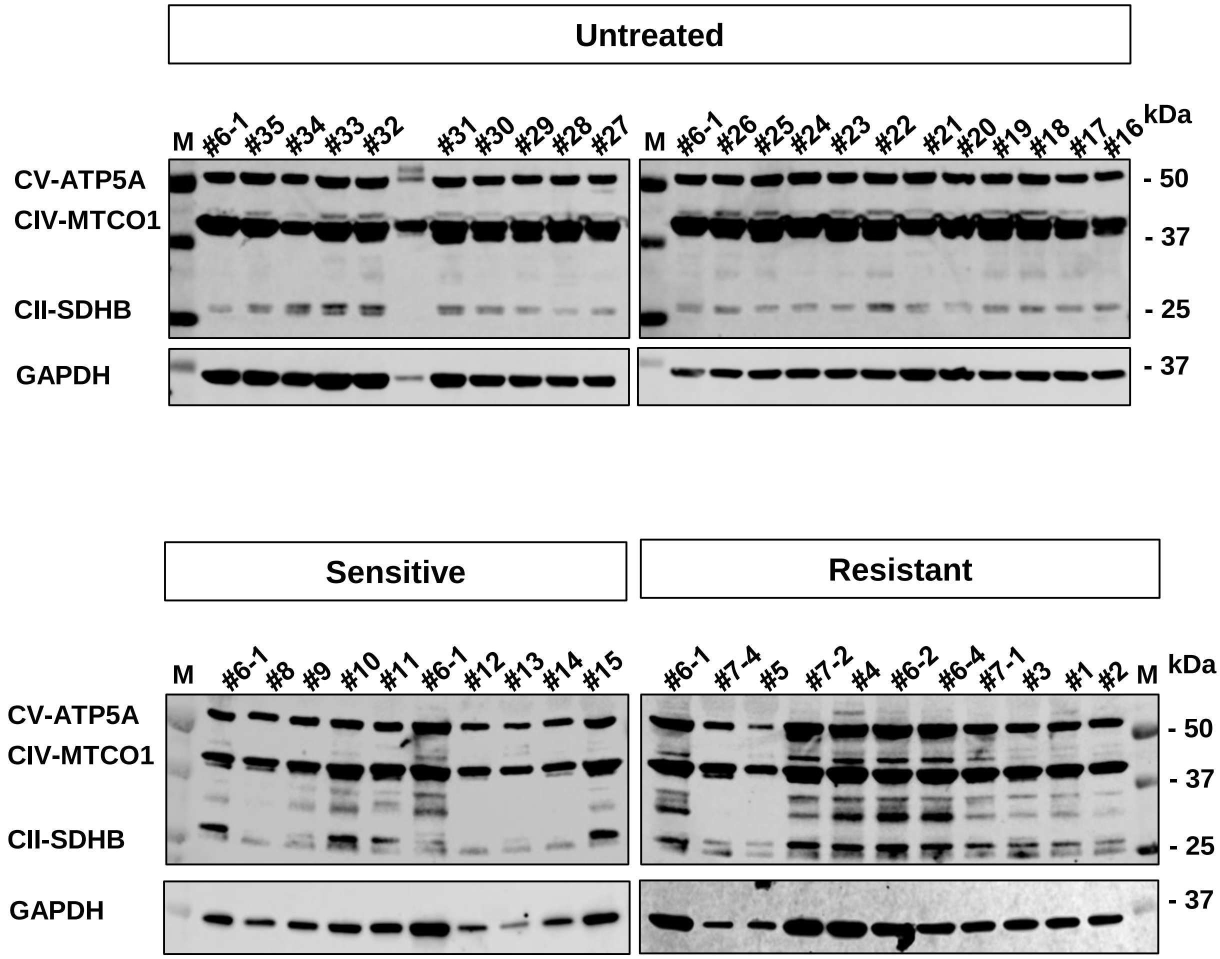

Figure S9. Immunoblots corresponding to Figure 7C showing OXPHOS proteins in 39 GIST tumors. Imatinib untreated samples are shown at the top and sensitive and resistant imatinib treated samples below. Molecular weight markers (M) are included in all immunoblots and the size markers are given to the right in kiloDaltons (kDa). Sample #6-1 was used as a reference for comparison between western blot membranes.
