# Supplementary material for: Heterogeneity of Metabolic Vulnerability in Imatinib-Resistant Gastrointestinal Stromal Tumor
Source: Cells. 2020 May 26;9(6):1333. doi: 10.3390/cells9061333 (PMC7348861; doi:10.3390/cells9061333)
Supplement: Supplementary file 1 [file cells-09-01333-s001.zip › cells-779087-supplementary/suppl-proof/Table S1.pdf]

Table S1. Clinical features, mutation status and imatinib treatment for the 35 GIST cases studied.

Case no.	Gender / age (yr)	Mutations		Tumor sample	Tumor size (cm)	Preoperative imatinib	
		KIT	*PDGFRA*			Period	Response at op
GIST1	M / 39	MUT	wt	Primary	5	36 m	Resistant
GIST2	F / 39	wt	MUT	Recurrent	2	15 m	Resistant
GIST3	M / 58	wt	MUT	Primary	16	3 m	Resistant
GIST4	F / 59	MUT	wt	Recurrent	3.7	>96 m	Resistant
GIST5	M / 33	MUT	wt	Recurrent	10	18 m	Resistant
GIST6*	F / 44	MUT	wt	Recurrent	4.5-30	10 m	Resistant
GIST7*	M / 46	MUT	wt	Primary	1-9.3	>48 m	Resistant
GIST8	M / 50	MUT	wt	Primary	5	12 m	Sensitive
GIST9	F / 54	MUT	wt	Primary	12	12 m	Sensitive
GIST10	F / 71	MUT	wt	Primary	6	6 m	Sensitive
GIST11	M / 67	wt	MUT	Primary	17	6 m	Sensitive
GIST12	M / 24	wt	wt	Primary	10	2 m	Sensitive
GIST13	F / 51	MUT	wt	Primary	6	18 m	Sensitive
GIST14	M / 35	MUT	wt	Primary	3	10 m	Sensitive
GIST15	F / 59	MUT	wt	Primary	7	5 m	Sensitive
GIST16	M / 75	MUT	wt	Recurrent	multiple	no	Untreated
GIST17	F / 72	MUT	wt	Primary	5.4	no	Untreated
GIST18	F / 57	MUT	wt	Recurrent	multiple	no	Untreated
GIST19	F / 56	MUT	wt	Primary	12	no	Untreated
GIST20	M / 49	MUT	wt	Primary	8.5	no	Untreated
GIST21	M / 55	MUT	wt	Primary	4	no	Untreated
GIST22	F / 54	MUT	wt	Primary	22	no	Untreated
GIST23	F / 73	MUT	wt	Primary	10	no	Untreated
GIST24	M / 69	MUT	wt	Primary	6.7	no	Untreated
GIST25	M / 82	MUT	wt	Primary	8	no	Untreated
GIST26	F / 70	MUT	wt	Recurrent	3	no	Untreated
GIST27	M / 63	MUT	wt	Recurrent	7	no	Untreated
GIST28	M / 70	MUT	wt	Primary	6	no	Untreated
GIST29	M / 57	MUT	wt	Recurrent	6	no	Untreated
GIST30	F / 79	MUT	wt	Primary	10.5	no	Untreated
GIST31	F / 65	MUT	wt	Primary	19	no	Untreated
GIST32	F / 36	MUT	wt	Primary	26	no	Untreated
GIST33	M / 71	MUT	wt	Primary	10	no	Untreated
GIST34	M / 65	MUT	wt	Primary	7	no	Untreated
GIST35	F / 52	MUT	wt	Primary	3	no	Untreated

*GIST6 and GIST7 had multiple tumors

yr, year; m, month; M, male; F, female; MUT, mutated; wt, wild-type; op, operation
