# Supplementary material for: Heterogeneity of Metabolic Vulnerability in Imatinib-Resistant Gastrointestinal Stromal Tumor
Source: Cells. 2020 May 26;9(6):1333. doi: 10.3390/cells9061333 (PMC7348861; doi:10.3390/cells9061333)
Supplement: Supplementary file 1 [file cells-09-01333-s001.zip › cells-779087-supplementary/suppl-proof/Table S2.pdf]

Table S2. Short tandem repeat profiles of the 5 human GIST cell lines in this study

	GIST 882		GIST 882R		GIST T1		GIST T1R		GIST 48	
**Locus**	**Al 1**	**Al 2**	**Al 1**	**Al 2**	**Al 1**	**Al 2**	**Al 1**	**Al 2**	**Al 1**	**Al 2**
D8S1179	12	12	12	12	13	13	13	13	13	13
D21S11	29	31.2	29	31.2	29	31	29	31	29	29
D7S820	10	15	10	15	11	12	11	12	9	12
CSF1PO	11	11	11	11	10	12	10	12	11	12
D3S1358	15	15	15	15	15	15	15	15	14	17
TH01	9	9	9	9	7	9	7	9	6	6
D13S317	12	12	12	12	11	11	11	11	13	13
D16S539	11	11	11	11	11	13	11	13	13	14
D2S1338	18	26	18	26	17	26	17	26	16	19
D19S433	13	15	13	15	14	15	14	15	13	17.2
vWA	16	17	16	17	14	14	14	14	14	15
TPOX	11	11	11	11	11	11	11	11	8	11
D18S51	14	14	14	14	14	14	14	14	17	18
AMEL	X	Y	X	Y	X	X	X	X	X	Y
D5S818	12	12	12	12	10	10	10	10	11	11
FGA	23	23	23	23	22	23	22	23	20	20

Al 1, Allele 1; Al 2, Allele 2
