# Supplementary material for: Heterogeneity of Metabolic Vulnerability in Imatinib-Resistant Gastrointestinal Stromal Tumor
Source: Cells. 2020 May 26;9(6):1333. doi: 10.3390/cells9061333 (PMC7348861; doi:10.3390/cells9061333)
Supplement: Supplementary file 1 [file cells-09-01333-s001.zip › cells-779087-supplementary/suppl-proof/Table S3.pdf]

Table S3. Differentially expressed genes for OXPHOS and glycolysis gene sets between Cluster I and II

Gene symbol	Gene title	Log Fold change	*p*-value	False discovery rates
OXPHOS gene set				
Overexpressed in Cluster II vs Cluster I				
NDUFA4	NDUFA4, mitochondrial complex associated	0.531	0.000	**0.018**
COX7B	cytochrome c oxidase subunit 7B	0.657	0.000	**0.018**
NDUFA3	NADH:ubiquinone oxidoreductase subunit A3	0.624	0.001	**0.018**
NDUFB7	NADH:ubiquinone oxidoreductase subunit B7	0.630	0.001	**0.018**
COX5B	cytochrome c oxidase subunit 5B	0.789	0.001	**0.019**
UQCRC2	ubiquinol-cytochrome c reductase core protein II	0.524	0.001	**0.019**
NDUFA9	NADH:ubiquinone oxidoreductase subunit A9	0.528	0.003	**0.055**
NDUFS7	NADH:ubiquinone oxidoreductase core subunit S7	0.320	0.003	**0.055**
COX6B1	cytochrome c oxidase subunit 6B1	0.476	0.004	**0.055**
NDUFS1	NADH:ubiquinone oxidoreductase core subunit S1	0.603	0.004	**0.055**
COX5A	cytochrome c oxidase subunit 5A	0.463	0.005	**0.057**
NDUFA8	NADH:ubiquinone oxidoreductase subunit A8	0.55	0.006	**0.065**
NDUFB3	NADH:ubiquinone oxidoreductase subunit B3	0.650	0.006	**0.065**
COX11	COX11, cytochrome c oxidase copper chaperone	0.437	0.007	**0.065**
NDUFA11	NADH:ubiquinone oxidoreductase subunit A11	0.340	0.007	**0.065**
SDHC	succinate dehydrogenase complex subunit C	0.362	0.008	**0.068**
COX7A2L	cytochrome c oxidase subunit 7A2 like	0.699	0.008	**0.070**
NDUFA1	NADH:ubiquinone oxidoreductase subunit A1	0.278	0.010	**0.075**
NDUFA7	NADH:ubiquinone oxidoreductase subunit A7	0.341	0.011	**0.079**
ATP6V0E1	ATPase H+ transporting V0 subunit e1	0.433	0.013	**0.089**
UQCRQ	ubiquinol-cytochrome c reductase complex III subunit VII	0.483	0.015	**0.090**
NDUFAB1	NADH:ubiquinone oxidoreductase subunit AB1	0.373	0.016	**0.090**
COX4I2	cytochrome c oxidase subunit 4I2	0.366	0.016	**0.090**
COX7A1	cytochrome c oxidase subunit 7A1	0.731	0.016	**0.090**
NDUFB5	NADH:ubiquinone oxidoreductase subunit B5	0.397	0.020	**0.104**
UQCRFS1	ubiquinol-cytochrome c reductase, Rieske iron-sulfur polypeptide 1	0.378	0.022	**0.106**
ATP6V1E2	ATPase H+ transporting V1 subunit E2	0.468	0.025	**0.114**
SDHB	succinate dehydrogenase complex iron sulfur subunit B	0.314	0.027	**0.114**
CYC1	cytochrome c1	0.547	0.027	**0.114**
NDUFS2	NADH:ubiquinone oxidoreductase core subunit S2	0.393	0.034	**0.132**
COX6A1	cytochrome c oxidase subunit 6A1	0.231	0.036	**0.134**
UQCR11	ubiquinol-cytochrome c reductase, complex III subunit XI	0.265	0.038	**0.134**
COX7C	cytochrome c oxidase subunit 7C	0.455	0.038	**0.134**
COX4I1	cytochrome c oxidase subunit 4I1	0.245	0.038	**0.134**
SDHA	succinate dehydrogenase complex flavoprotein subunit A	0.455	0.040	**0.137**
COX15	COX15, cytochrome c oxidase assembly homolog	0.290	0.041	**0.137**
NDUFV2	NADH:ubiquinone oxidoreductase core subunit V2	0.492	0.041	**0.137**
UQCRC1	ubiquinol-cytochrome c reductase core protein I	0.349	0.046	**0.145**
NDUFV1	NADH:ubiquinone oxidoreductase core subunit V1	0.245	0.059	**0.175**
COX7B2	cytochrome c oxidase subunit 7B2	0.179	0.059	**0.175**
NDUFA2	NADH:ubiquinone oxidoreductase subunit A2	0.287	0.062	**0.175**
COX6A2	cytochrome c oxidase subunit 6A2	0.399	0.062	**0.175**

NDUFA10	NADH:ubiquinone oxidoreductase subunit A10	0.163	0.063	**0.175**
SDHD	succinate dehydrogenase complex subunit D	0.238	0.076	**0.190**
NDUFB10	NADH:ubiquinone oxidoreductase subunit B10	0.249	0.077	**0.190**
NDUFA6	NADH:ubiquinone oxidoreductase subunit A6	0.407	0.085	0.205
NDUFV3	NADH:ubiquinone oxidoreductase subunit V3	0.307	0.093	0.215
NDUFB2	NADH:ubiquinone oxidoreductase subunit B2	0.264	0.094	0.215
ATP6AP1	ATPase H+ transporting accessory protein 1	0.239	0.108	0.238
NDUFB9	NADH:ubiquinone oxidoreductase subunit B9	0.348	0.134	0.282
NDUFB6	NADH:ubiquinone oxidoreductase subunit B6	0.225	0.140	0.291
UQCRB	ubiquinol-cytochrome c reductase binding protein	0.415	0.144	0.296
COX8A	cytochrome c oxidase subunit 8A	0.289	0.148	0.300
NDUFC1	NADH:ubiquinone oxidoreductase subunit C1	0.238	0.153	0.306
ATP6V1C1	ATPase H+ transporting V1 subunit C1	0.313	0.179	0.345
ATP6V0B	ATPase H+ transporting V0 subunit b	0.298	0.212	0.384
ATP6V1G1	ATPase H+ transporting V1 subunit G1	0.270	0.214	0.384
NDUFS3	NADH:ubiquinone oxidoreductase core subunit S3	0.296	0.234	0.409
COX6C	cytochrome c oxidase subunit 6C	0.148	0.287	0.467
ATP6V0A4	ATPase H+ transporting V0 subunit a4	0.066	0.426	0.646
ATP6V1A	ATPase H+ transporting V1 subunit A	0.126	0.436	0.653
PPA1	pyrophosphatase (inorganic) 1	0.210	0.456	0.659
ATP6V0E2	ATPase H+ transporting V0 subunit e2	0.265	0.458	0.659
COX6B2	cytochrome c oxidase subunit 6B2	0.073	0.465	0.659
COX7A2	cytochrome c oxidase subunit 7A2	0.156	0.511	0.699
ATP6V1D	ATPase H+ transporting V1 subunit D	0.092	0.575	0.741
NDUFS4	NADH:ubiquinone oxidoreductase subunit S4	0.075	0.647	0.783
NDUFA4L2	NDUFA4, mitochondrial complex associated like 2	0.083	0.706	0.841
ATP4A	ATPase H+/K+ transporting alpha subunit	0.038	0.775	0.896
NDUFS6	NADH:ubiquinone oxidoreductase subunit S6	0.042	0.843	0.946
UQCR10	ubiquinol-cytochrome c reductase, complex III subunit X	0.027	0.892	0.967
COX10	COX10, heme A:farnesyltransferase cytochrome c oxidase assembly factor	0.015	0.905	0.967
PPA2	pyrophosphatase (inorganic) 2	0.013	0.933	0.990
ATP6V1G3	ATPase H+ transporting V1 subunit G3	0.005	0.959	0.992
ATP6V1G2	ATPase H+ transporting V1 subunit G2	0.016	0.969	0.992
NDUFB8	NADH:ubiquinone oxidoreductase subunit B8	0.004	0.971	0.992
ATP4B	ATPase H+/K+ transporting beta subunit	0.001	0.993	0.995
Underexpressed in Cluster II vs Cluster I				
ATP6V0A1	ATPase H+ transporting V0 subunit a1	-0.657	0.005	**0.057**
ATP6V1H	ATPase H+ transporting V1 subunit H	-0.387	0.027	**0.114**
ATP12A	ATPase H+/K+ transporting non-gastric alpha2 subunit	-0.204	0.045	**0.145**
ATP6V0D2	ATPase H+ transporting V0 subunit d2	-0.200	0.064	**0.175**
ATP6V1B2	ATPase H+ transporting V1 subunit B2	-0.419	0.118	0.256
NDUFB4	NADH:ubiquinone oxidoreductase subunit B4	-0.112	0.217	0.384
COX8C	cytochrome c oxidase subunit 8C	-0.088	0.243	0.416
COX17	COX17, cytochrome c oxidase copper chaperone	-0.144	0.392	0.599
LHPP	phospholysine phosphohistidine inorganic pyrophosphate phosphatase	-0.162	0.475	0.665
ATP6V0D1	ATPase H+ transporting V0 subunit d1	-0.116	0.534	0.713
NDUFB1	NADH:ubiquinone oxidoreductase subunit B1	-0.110	0.535	0.713
NDUFA5	NADH:ubiquinone oxidoreductase subunit A5	-0.051	0.638	0.783

ATP6V1E1	ATPase H+ transporting V1 subunit E1	-0.130	0.641	0.783
TCIRG1	T-cell immune regulator 1, ATPase H+ transporting V0 subunit a3	-0.161	0.642	0.783
ATP6V1C2	ATPase H+ transporting V1 subunit C2	-0.019	0.825	0.939
ATP6V0A2	ATPase H+ transporting V0 subunit a2	-0.029	0.840	0.946
ATP6V1B1	ATPase H+ transporting V1 subunit B1	-0.015	0.890	0.967
ATP6V0C	ATPase H+ transporting V0 subunit c	-0.001	0.995	0.995
Glycolysis gene set				
Overexpressed in Cluster II vs Cluster I				
ALDH1B1	aldehyde dehydrogenase 1 family member B1	0.590	0.001	**0.018**
PDHB	pyruvate dehydrogenase (lipoamide) beta	0.546	0.011	**0.080**
PGK1	phosphoglycerate kinase 1	0.592	0.014	**0.090**
DLAT	dihydrolipoamide S-acetyltransferase	0.618	0.018	**0.098**
PGK2	phosphoglycerate kinase 2	0.213	0.021	**0.106**
ALDOA	aldolase, fructose-bisphosphate A	0.415	0.024	**0.114**
BPGM	bisphosphoglycerate mutase	0.697	0.029	**0.117**
GPI	glucose-6-phosphate isomerase	0.523	0.054	**0.170**
LDHB	lactate dehydrogenase B	0.363	0.062	**0.175**
DLD	dihydrolipoamide dehydrogenase	0.424	0.067	**0.177**
GCK	glucokinase	0.522	0.073	**0.190**
PGAM1	phosphoglycerate mutase 1	0.348	0.078	**0.190**
ALDH2	aldehyde dehydrogenase 2 family (mitochondrial)	0.457	0.108	0.238
LDHC	lactate dehydrogenase C	0.346	0.167	0.330
LDHAL6A	lactate dehydrogenase A like 6A	0.271	0.173	0.337
PGM1	phosphoglucomutase 1	0.272	0.182	0.347
PDHA2	pyruvate dehydrogenase (lipoamide) alpha 2	0.125	0.191	0.359
ENO1	enolase 1	0.342	0.208	0.381
FBP2	fructose-bisphosphatase 2	0.115	0.253	0.428
PDHA1	pyruvate dehydrogenase (lipoamide) alpha 1	0.233	0.255	0.428
GALM	galactose mutarotase	0.162	0.282	0.467
HK2	hexokinase 2	0.226	0.285	0.467
LDHA	lactate dehydrogenase A	0.295	0.300	0.483
ADH5	alcohol dehydrogenase 5 (class III), chi polypeptide	0.201	0.310	0.494
ALDOC	aldolase, fructose-bisphosphate C	0.343	0.342	0.529
TPI1	triosephosphate isomerase 1	0.179	0.449	0.659
PKLR	pyruvate kinase, liver and RBC	0.062	0.463	0.659
HK3	hexokinase 3	0.102	0.545	0.720
ENO2	enolase 2	0.418	0.567	0.741
PKM	pyruvate kinase, muscle	0.127	0.594	0.759
PFKM	phosphofructokinase, muscle	0.129	0.604	0.766
ALDH3A1	aldehyde dehydrogenase 3 family member A1	0.045	0.615	0.773
PFKP	phosphofructokinase, platelet	0.115	0.647	0.783
ADH1C	alcohol dehydrogenase 1C (class I), gamma polypeptide	0.207	0.740	0.875
ADH1A	alcohol dehydrogenase 1A (class I), alpha polypeptide	0.046	0.746	0.875
ALDH3A2	aldehyde dehydrogenase 3 family member A2	0.048	0.809	0.928
GAPDH	glyceraldehyde-3-phosphate dehydrogenase	0.039	0.865	0.960
PCK2	phosphoenolpyruvate carboxykinase 2, mitochondrial	0.038	0.868	0.960
PGAM2	phosphoglycerate mutase 2	0.054	0.902	0.967
ENO3	enolase 3	0.006	0.964	0.992

ALDOB	aldolase, fructose-bisphosphate B	0.002	0.973	0.992
ALDH9A1	aldehyde dehydrogenase 9 family member A1	0.003	0.987	0.995
Underexpressed in Cluster II vs Cluster I				
PFKL	phosphofructokinase, liver type	-0.706	0.031	**0.126**
ALDH1A3	aldehyde dehydrogenase 1 family member A3	-0.981	0.065	**0.176**
ALDH3B1	aldehyde dehydrogenase 3 family member B1	-0.169	0.076	**0.190**
G6PC2	glucose-6-phosphatase catalytic subunit 2	-0.218	0.091	0.215
ACSS1	acyl-CoA synthetase short-chain family member 1	-0.169	0.105	0.238
ADH1B	alcohol dehydrogenase 1B (class I), beta polypeptide	-0.690	0.127	0.271
ACSS2	acyl-CoA synthetase short-chain family member 2	-0.154	0.195	0.362
LDHAL6B	lactate dehydrogenase A like 6B	-0.099	0.240	0.416
HK1	hexokinase 1	-0.267	0.329	0.519
G6PC	glucose-6-phosphatase catalytic subunit	-0.126	0.340	0.529
ALDH3B2	aldehyde dehydrogenase 3 family member B2	-0.053	0.442	0.657
PGM2	phosphoglucomutase 2	-0.120	0.477	0.665
PCK1	phosphoenolpyruvate carboxykinase 1	-0.160	0.491	0.678
FBP1	fructose-bisphosphatase 1	-0.226	0.518	0.703
ADH6	alcohol dehydrogenase 6 (class V)	-0.044	0.570	0.741
ALDH7A1	aldehyde dehydrogenase 7 family member A1	-0.068	0.682	0.818
AKR1A1	aldo-keto reductase family 1 member A1	-0.051	0.752	0.876
ADH7	alcohol dehydrogenase 7 (class IV), mu or sigma polypeptide	-0.009	0.899	0.967
ADH4	alcohol dehydrogenase 4 (class II), pi polypeptide	-0.006	0.940	0.991

Differentially expressed genes (false discovery rate <0.2) used for clustering analysis are indicated in bold
